# Reverse diauxie phenotype in *Pseudomonas aeruginosa* biofilm revealed by exometabolomics and label-free proteomics

**DOI:** 10.1038/s41522-019-0104-7

**Published:** 2019-10-25

**Authors:** Yeni P. Yung, S. Lee McGill, Hui Chen, Heejoon Park, Ross P. Carlson, Luke Hanley

**Affiliations:** 1 Department of Chemistry, University of Illinois at Chicago, Chicago, IL 60607 USA; 2 Center for Biofilm Engineering, Montana State University, Bozeman, MT 59717 USA; 3 Research Resources Center, University of Illinois at Chicago, Chicago, IL 60607 USA

**Keywords:** Biofilms, Bacteriology

## Abstract

Microorganisms enhance fitness by prioritizing catabolism of available carbon sources using a process known as carbon catabolite repression (CCR). Planktonically grown *Pseudomonas aeruginosa* is known to prioritize the consumption of organic acids including lactic acid over catabolism of glucose using a CCR strategy termed “reverse diauxie.” *P. aeruginosa* is an opportunistic pathogen with well-documented biofilm phenotypes that are distinct from its planktonic phenotypes. Reverse diauxie has been described in planktonic cultures, but it has not been documented explicitly in *P. aeruginosa* biofilms. Here a combination of exometabolomics and label-free proteomics was used to analyze planktonic and biofilm phenotypes for reverse diauxie. *P. aeruginosa* biofilm cultures preferentially consumed lactic acid over glucose, and in addition, the cultures catabolized the substrates completely and did not exhibit the acetate secreting “overflow” metabolism that is typical of many model microorganisms. The biofilm phenotype was enabled by changes in protein abundances, including lactate dehydrogenase, fumarate hydratase, GTP cyclohydrolase, L-ornithine N(5)-monooxygenase, and superoxide dismutase. These results are noteworthy because reverse diauxie-mediated catabolism of organic acids necessitates a terminal electron acceptor like O_2_, which is typically in low supply in biofilms due to diffusion limitation. Label-free proteomics identified dozens of proteins associated with biofilm formation including 16 that have not been previously reported, highlighting both the advantages of the methodology utilized here and the complexity of the proteomic adaptation for *P. aeruginosa* biofilms. Documenting the reverse diauxic phenotype in *P. aeruginosa* biofilms is foundational for understanding cellular nutrient and energy fluxes, which ultimately control growth and virulence.

## Introduction

*Pseudomonas aeruginosa* is an opportunistic pathogen capable of forming biofilms and is commonly associated with chronic wounds, cystic fibrosis lungs, and medical device-related infections.^1–3^ *P. aeruginosa* has been widely studied for its ability to acclimate to a broad range of stresses, including antimicrobial exposure, host cell interactions, and surface chemistries.^4–6^ Microorganisms, including *P. aeruginosa*, maximize fitness by prioritizing catabolism of available carbon sources while repressing catabolism of non-preferred carbon sources, a regulatory process known as carbon catabolite repression (CCR).^7–10^ CCR is one of the most important global regulatory systems in microorganisms and is central to nutrient acquisition for growth and cellular energy generation as well as for virulence-associated processes like quorum sensing and biofilm formation. Understanding microorganism-specific CCR processes provides foundational understanding of ecological strategies for extracting nutrients and energy from complex environments, such as chronic wounds. CCR strategies and regulatory networks of widely studied microorganisms like *Escherichia coli* and *Bacillus subtilis* have become paradigms of substrate preference where glucose is catabolized prior to other sugars like lactose or organic acids. Many *Pseudomonads* including *P. aeruginosa* have evolved a markedly different CCR-based preference for substrates. *Pseudomonads* prioritize the consumption of organic acids including acetic, citric and lactic acids over catabolism of glucose using a CCR strategy that has been termed “reverse diauxie” or “reverse catabolite repression control.”^11,12^ *Pseudomonad* reverse diauxie is controlled by global regulators including RNA-binding protein (Hfq) and catabolite repression control protein (Crc) and a small non-coding RNA (CrcZ) as compared to the phosphotransferase system studied in *E. coli* and *B. subtilis*.^13–16^

Reverse diauxie has been described in *P. aeruginosa* planktonic cultures,^11,12^ but to the best knowledge of the authors, a reverse diauxie phenotype has not been explicitly described in a biofilm phenotype based on exometabolomics and proteomics. This distinction is significant because biofilm phenotypes are well known to differ from planktonic phenotypes due to a myriad of factors including nutrient diffusion limitation, gradients in chemical environments as well as distinct biofilm regulatory processes.^17–25^ Directly measuring the reverse diauxic phenotype in *P. aeruginosa* biofilms is essential for understanding cellular nutrient and energy fluxes, which ultimately control energy metabolism, growth, and virulence.

Proteomics can better quantify phenotypes, like the acclimation to nutrient availability, than transcriptomics by identifying the actual proteins in which the cultures have invested scarce anabolic nutrients.^26^ Rich data obtained from proteomics including the abundance of enzymes and other proteins can inform the study of complex biological systems.^20,22,27–30^ For example, proteomic strategies have been used to directly link enzyme abundance changes to metabolic fluxes^31^ and differentiate exudates from acute and chronic wounds of human skin.^32^

*P. aeruginosa* proteomic studies have been reported using two-dimensional gel electrophoresis with mass spectrometry (MS).^19–22,24^ This offline approach can separate thousands of proteins but is low throughput.^20,22^ Alternatively, shotgun proteomics uses online liquid chromatography–MS/MS (LC-MS/MS) to simultaneously separate, identify, and quantify proteins from whole-cell lysates with high-resolution mass analyzers such as an Orbitrap or quadrupole time-of-flight.^33^ Shotgun proteomics can rapidly screen peptides, making it advantageous for targeted and untargeted analyses with better sample throughput and can be used with labeling or label-free strategies.^34,35^

The present study uses exometabolomics and proteomics to document reverse diauxie during biofilm growth of a *P. aeruginosa* strain isolated from a chronic wound. A combination of exometabolomics and label-free proteomics provides evidence of reverse diauxie in biofilms highlighting the expressed metabolic strategy even in the presence of biofilm-associated mass transfer limitation of metabolites, including oxygen. This study fills a phenotype gap in the literature of *P. aeruginosa* biofilm metabolism and identifies 16 proteins that have not been reported previously to be associated with biofilm formation. It also provides ecological insight necessary for interpreting biofilm cultures as well as devising a rational basis for the computational modeling of *P. aeruginosa* during biofilm growth.^36^

## Results

### Exometabolomics in support of reverse diauxie in *P. aeruginosa* biofilm cultures

Planktonic and biofilm cultures of *P. aeruginosa* were grown on glucose-containing, CSP medium or lactate-supplemented, CSP (LCSP) medium, where CSP is a chemically defined and nutritionally complete growth medium (see “Methods”). Biofilm cultures of *P. aeruginosa* were grown under no-shear conditions on polyester membranes in contact with liquid phase medium, permitting the temporal measurement of substrate and byproduct concentrations.^37^

**Fig. 1:**
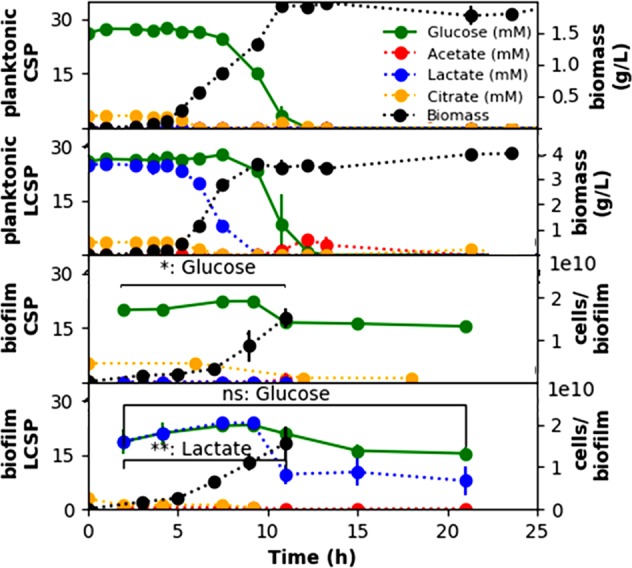
Time-dependent *P. aeruginosa* culture properties as a function of growth medium (CSP, LCSP) and cultivation method (planktonic, biofilm). Metabolite concentrations are presented for glucose, acetate, lactate, and citrate; biomass levels were also plotted on the right axes. Citrate was consumed preferentially to lactate, which was consumed preferentially to glucose in both planktonic and biofilm cultures. Single (*) and double (**) asterisks indicate statistically significant changes in metabolite concentration over the indicated time range. ns = not significant change in metabolite concentration over the indicated time range

**Fig. 2:**
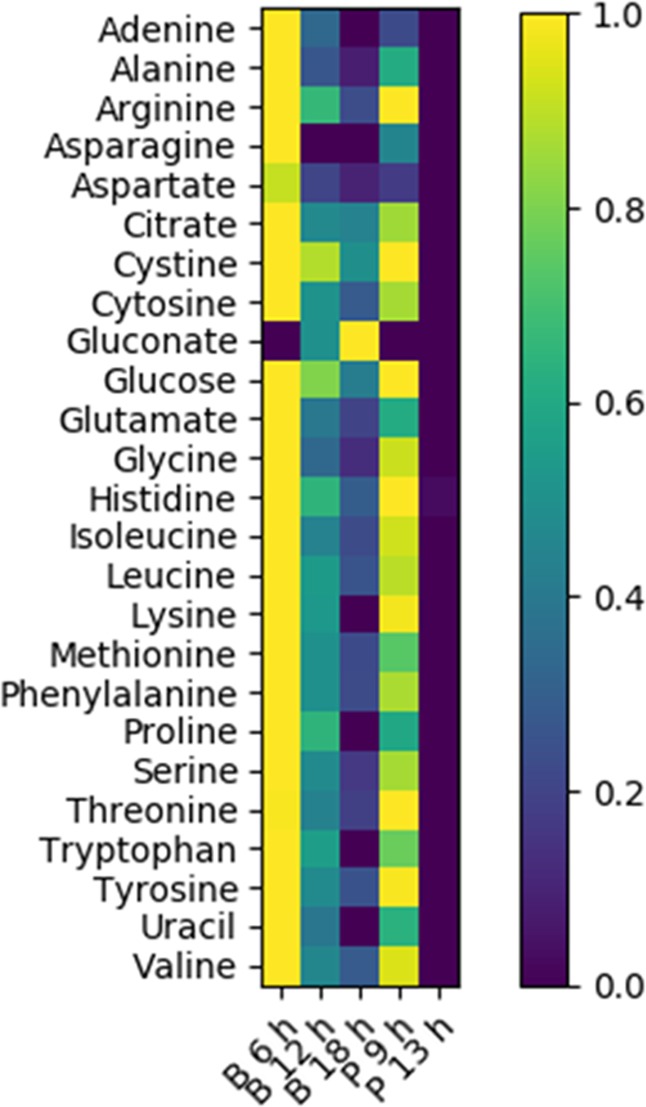
Summary of NMR metabolomics analysis of CSP-grown *P. aeruginosa* biofilm and planktonic cultures. Heat map quantifies fractional changes in metabolite concentration (mM) from initial conditions (*t* = 0 h) with the exception of gluconate, which was produced by the biofilm cultures and normalized to final concentration (0.4 mM). Values vary from 1 to 0. CSP-grown biofilm cultures demonstrated a preferential catabolism of amino acids followed by citrate and finally glucose. B indicates biofilm culture grown on CSP, P indicates planktonic culture grown on CSP, numbers refer to culturing time in hours (h). Errors are standard deviations

Exometabolomic analyses were performed using high-performance liquid chromatography (HPLC) and proton nuclear magnetic resonance (NMR) and are shown in Figs 1 and 2, respectively. Biomass levels measured as a function of time are shown in Fig. 1. Planktonic cultures had higher specific growth rates than the biofilm cultures, presumably due to mass transfer limitations and substrate gradients within the biofilm (see below). Nevertheless, the specific growth rates were not significantly different between the two medium formulations (CSP and LCSP) during planktonic or biofilm growth, as shown in Table 1. The presence of lactate in LCSP medium resulted in higher, final biomass titers for the planktonic cultures, but this trend was not observed in biofilm cultures.

**Table 1:** Summary of *P. aeruginosa-*specific growth rates and doubling times for planktonic and biofilm cultures grown on either CSP or LCSP medium (see “Methods” for error analysis)

	Planktonic		Biofilm	
Medium	CSP	LCSP	CSP	LCSP
Growth rate (h^−1^)	0.87 ± 0.05	0.88 ± 0.02	0.37 ± 0.09	0.31 ± 0.05
Doubling time (h)	0.8 ± 0.05	0.8 ± 0.02	1.9 ± 0.54	2.29 ± 0.39

Both the CSP and LCSP media contained citrate, which was added as an iron chelator. Citrate was observed here to be consumed preferentially under all conditions as compared to glucose or lactate, consistent with documented reverse diauxie.^13^ Furthermore, lactate was consumed preferentially over glucose by both planktonic and biofilm cultures (Fig. 1). LCSP-grown cultures did not catabolize significant amounts of glucose while lactate was present. Glucose was catabolized by the planktonic cultures after the exhaustion of lactate. The CSP biofilm cultures consumed approximately half of the present lactate and did not catabolize a significant amount of glucose.

Acetate is a commonly observed overflow metabolite secreted by classic CCR-utilizing microorganisms, including *E. coli* and *B. subtilis*.^38–40^ However, neither the planktonic- nor biofilm-grown *P. aeruginosa* cultures secreted large quantities of organic byproducts like acetate, as shown in Fig. 2. Planktonic, LCSP-grown cultures produced measurable (~5 mM) acetate during the exhaustion of glucose, but initial data indicate that it was quickly consumed as a substrate. Acetate concentrations were near the detection limit (0.01 mM) for the other culturing conditions. This suggests not only a greater yield of available energy than is observed in overflow metabolisms but also a greater investment of anabolic nutrients into respiratory catabolic pathways. *Pseudomonads* have been observed to release gluconate or 2-ketogluconate during glucose catabolism.^41^ Initial NMR analysis quantified small concentrations of gluconate (~0.4 mM) during the later time points in Fig. 2, indicating that CSP-grown biofilms provide evidence of glucose catabolism. However, this metabolite was not observed in planktonic cultures. A summary of the comprehensive NMR exometabolomics analysis is also shown in Fig. 2 and the complete set of NMR data is provided as Supplementary Table 1.

**Fig. 3:**
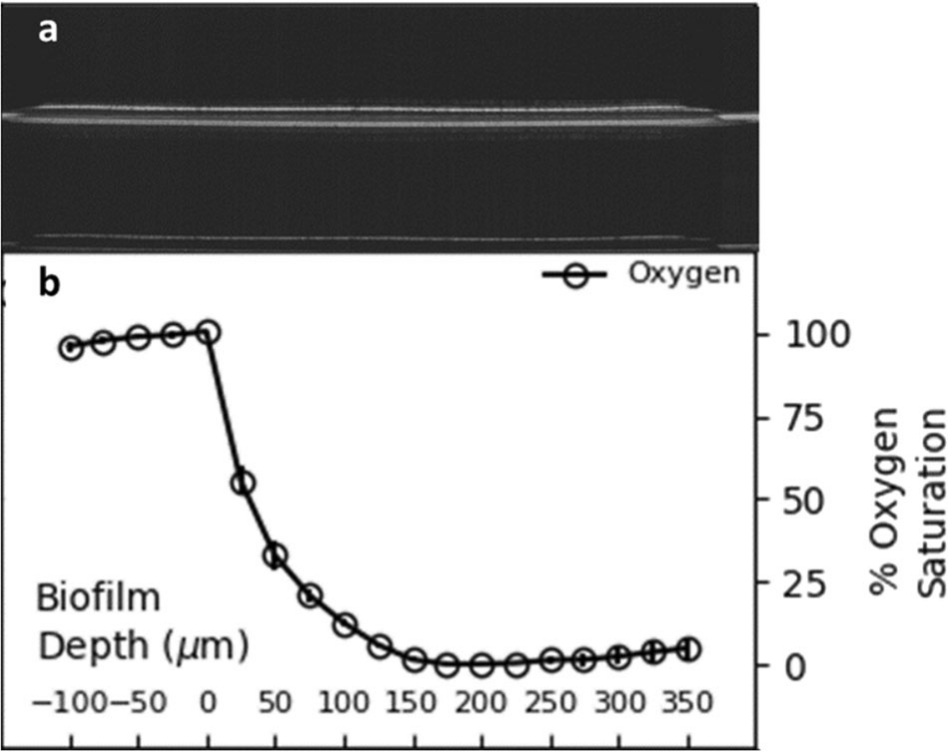
**a** Optical coherence tomography (OCT) cross-section image of a *P. aeruginosa* biofilm, where average biofilm height is 0.010 ± 0.001 cm (standard deviation). **b** Oxygen saturation gradient measured by microelectrode within the biofilm showing an exponential decrease in oxygen saturation resulting in an anoxic region

The biofilms were oxygen-diffusion limited; the top ~150 μm of biofilm cultures had measurable oxygen concentrations while the oxygen concentration was below the detection limit in the bottom 200 μm, as shown in Fig. 3. Despite the large anoxic zone, the biofilm cultures did not secrete more than trace amounts of acetate. Analysis of optical coherence tomographic (OCT) images of 7-h biofilms found an average biofilm height of 0.010 ± 0.001 cm, an average biofilm diameter of 0.58 ± 0.07 cm, and an average biofilm volume of 0.0028 ± 0.0008 cm^3^. The biofilms analyzed by OCT had (2.3 ± 0.8) × 10^9^ colony-forming units/biofilm with average biomass concentration within the biofilm of 130 ± 10 g cell dry weight/L.

### Proteomics in support of reverse diauxie in *P. aeruginosa* biofilm cultures

**Fig. 4:**
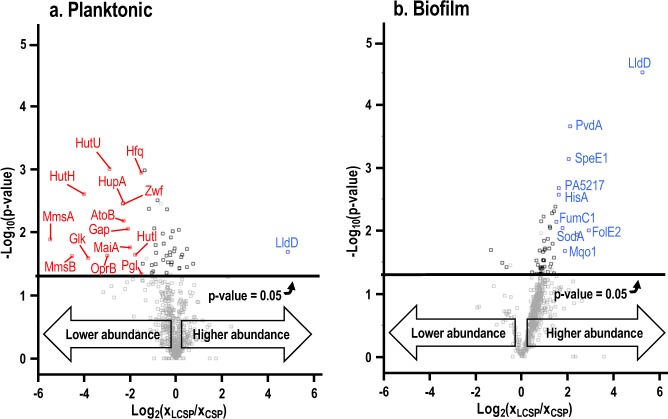
Differential protein abundance for *P. aeruginosa* cultures grown in LCSP vs. CSP medium. **a** Planktonic cultures. **b** Biofilm cultures. Samples collected at 11 h of growth. Proteins with statistically significant (*p* value < 0.05) changes in abundance are highlighted above horizontal black line. Proteins with |log_2_(*x*_LCSP_/*x*_CSP_)| > 1.5, where *x*_(L)CSP_ is protein abundance, are deemed as significantly lower or higher abundance and are annotated as red or blue points, respectively

Cell pellets from planktonic and biofilm cultures were collected after 11 h of growth and analyzed for proteomics. The 11-h time point permitted a uniform culturing time for analyzing all four cultures and corresponded with the later phase of lactate catabolism in the planktonic culture and the mid-phase of lactate catabolism in the biofilm culture. Untargeted, label-free quantitative processing of the mass spectral data set identified ~400 proteins collectively from all cultures studied here, with the entire list including Kyoto Encyclopedia of Genes and Genome (KEGG) IDs and protein descriptions provided as Supplementary Tables 2–5. Proteins were identified by either a minimum of two unique peptides or sequence coverage >10%, although >80% of all identified proteins met both criteria. Identified proteins were designated as having significantly higher or lower abundance based on quantitative proteomics (see “Methods” section for precise definition). Fifty proteins from the planktonic cultures displayed statistically significant differences in abundance in LCSP vs. CSP media at 11 h of growth, as shown in Fig. 4a. Fifty one proteins had different abundances for the analogous biofilm cultures, as shown in Fig. 4b. Proteomics identified multiple proteins that displayed statistically significant differences in abundance between compared growth media (LCSP vs. CSP) and culturing methods (biofilm vs. planktonic), as shown in Fig. 4 and Table 2. Supplementary Figs 3–6 graphically illustrate the *P. aeruginosa* central metabolism and highlight enzymes that were observed experimentally to display statistically significant changes in abundance.

**Table 2:** *P. aeruginosa* proteins with significantly higher (↑) and lower abundances (↓) for protein abundances for growth in LCSP vs. CSP medium: protein abbreviations, KEGG IDs, full protein names, number of unique peptides, overall sequence coverage, and log_2_(*x*_LCSP_/*x*_CSP_) where *x*_(L)CSP_ is protein abundance are provided for each protein (see “Methods” for error analysis)

Culture	Protein abbreviation	KEGG ID	Protein name	Unique peptides	Sequence coverage	Log_2_(*x*_LCSP_/*x*_CSP_)
Planktonic	LldD	PA4771	L-lactate dehydrogenase	14	44.4%	4.9 ↑
	MmsA	PA3570	Methylmalonate-semialdehyde dehydrogenase [acylating]	18	50.1%	−5.5 ↓
	MmsB	PA3569	3-Hydroxyisobutyrate dehydrogenase	9	52.7%	−4.5 ↓
	HutH	PA5098	Histidine ammonia-lyase	8	27.5%	−4.0 ↓
	Glk	PA3193	Glucokinase	2	12.1%	−3.8 ↓
	OprB	PA3186	Porin B	15	44.3%	−3.0 ↓
	HutU	PA5100	Urocanate hydratase	23	55.3%	−2.9 ↓
	Zwf	PA3183	Glucose-6-phosphate 1-dehydrogenase	18	53%	−2.3 ↓
	HupA	PA5348	DNA-binding protein HU-alpha	9	73.3%	−2.3 ↓
	AtoB	PA2001	Acetyl-CoA acetyltransferase	15	62.6%	−2.3 ↓
	Gap	PA3195	Glyceraldehyde 3-phosphate dehydrogenase	16	59.0%	−2.1 ↓
	MaiA	PA2007	Maleylacetoacetate isomerase	5	39.2%	−2.0 ↓
	HutI	PA5092	Imidazolonepropionase	3	11.2%	−1.8 ↓
	Pgl	PA3182	6-phosphogluconolactonase	9	52.1%	−1.5 ↓
	Hfq	PA4944	RNA-binding protein in *P. aeruginosa*	16	51.3%	−1.5 ↓
Biofilm	LldD	PA4771	L-lactate dehydrogenase	12	39.60%	5.3 ↑
	FolE2	PA5539	GTP cyclohydrolase	6	36.20%	2.9 ↑
	PvdA	PA2386	L-ornithine N(5)-monooxygenase	4	12.40%	2.1 ↑
	SpeE1	PA1687	Polyamine aminopropyltransferase 1	3	23.80%	2.0 ↑
	Mqo1	PA3452	Malate:quinone oxidoreductase 1 (probable)	4	11.10%	1.9 ↑
	SodA	PA4468	Superoxide dismutase [Mn]	3	25.10%	1.8 ↑
	PA5217	PA5217	Binding protein component of ABC iron transporter PA5217 (probable)	12	36.10%	1.6 ↑
	HisA	PA5141	1-(5-phosphoribosyl)-5-[(5-phosphoribosylamino) methylideneamino] imidazole-4-carboxamide isomerase	6	32.70%	1.6 ↑
	FumC1	PA4470	Fumarate hydratase	19	59.60%	1.5 ↑

Analysis of the proteins from the planktonic cultures demonstrated clear reverse diauxie patterns with higher abundance of the key lactate catabolizing enzyme L-lactate dehydrogenase (LldD) in LCSP-grown cultures. Lower abundances of glucose catabolism-associated proteins, including glucose transporter (OprB), glucokinase (Glk), and enzymes from the Entner–Doudoroff glycolysis pathway (Zwf, Pgl, and Gap), were also observed in LCSP. *P. aeruginosa* does not possess a complete Embden–Meyerhof–Parnas glycolysis pathway and instead uses the Entner–Doudoroff pathway.^42^ Biofilm cultures had similarly high abundances of LldD when grown in LCSP medium. However, no proteins with significantly lower abundances were observed in LCSP-grown biofilms, in contrast to what was observed with the planktonic cultures: this might have resulted from the averaging of phenotypes from the top to the bottom of the biofilms. Figure 3b supports this argument, as it confirms that the biofilm phenotype varies with nutrient gradients. Biofilm cultures grown on LCSP also displayed higher abundances of two tricarboxylic acid (TCA) cycle enzymes: fumarate hydratase (FumC1), involved in fumarate conversion to S-malate, and malate:quinone oxidoreductase 1 (Mqo1, probable assignment), involved in S-malate conversion to oxaloacetate. No published description of Mqo1 in *P. aeruginosa* was found, although the related enzyme Mqo2 was observed to be essential for *P. aeruginosa* growth in the presence of acetate and ethanol.^43^

Other catabolic pathways associated with components of the growth medium were repressed in the presence of lactate during planktonic growth. These included the decreased abundance of enzymes involved in the metabolism of histidine (HutH, HutI, and HutU), alanine, valine, leucine, and isoleucine (AtoB, MmsA, and MmsB) as well as aromatic amino acids (MaiA).^44^

Hfq, a global metabolism regulator associated with reverse diauxie, displayed significantly lower abundance in planktonic cultures grown with lactate. Repression of the Hfq-Crc complex by CrcZ, a small non-coding RNA, permits translation of genes encoding proteins for non-preferred substrates in *P. aeruginosa* strain PAO1.^16,45,46^ Table 3 shows five proteins with lower abundances in LCSP vs. CSP for planktonic cultivation and the one protein with higher abundance during biofilm cultivation whose transcripts were reported to be upregulated or downregulated by mutation of this Hfq-Crc-CrcZ CCR system.^46,47^ Furthermore, the HupA transcript in *P. aeruginosa* has been associated with stress acclimation,^48,49^ while HupA has a regulatory role in *E. coli* by increasing aggregation and adhesion.^50^ Notably, the catabolite repression control protein Crc was not observed here.^13–16^

**Table 3:** *P. aeruginosa* proteins with higher (↑) or lower abundances (↓) in LCSP vs. CSP for planktonic or biofilm cultures whose transcriptomes were reported to be upregulated (↑) or downregulated (↓) by mutation of Hfq-Crc-CrcZ carbon catabolite repression system

Protein	KEGG ID	Proteins in planktonic or biofilm (here)	Transcriptome			
			Hfq mutant^48^	Crc mutant^47^	Cbr mutant^47^	CrcZ mutant^47^
HupA	PA5348	↓/Planktonic	—	↑	—	↓
MmsA	PA3570	↓/Planktonic	—	↑	—	—
MmsB	PA3569	↓/Planktonic	↑	—	—	—
OprB	PA3186	↓/Planktonic	↑	—	—	—
Zwf	PA3183	↓/Planktonic	—	—	↑	—
MqoA	PA3452	↑/Biofilm	—	↑	↑	—

An additional ~300 proteins not displaying significant changes in abundance were observed and are also of note given their potential role in metabolism. These potentially metabolically relevant proteins with non-significant abundance differentials are listed in Supplementary Tables 2–5, which include all proteins observed here including those listed in Tables 2–4.

**Table 4:** *P. aeruginosa* proteins demonstrating statistically significant higher (↑) and lower abundances (↓) for biofilm vs. planktonic growth in both LCSP and CSP media

Protein	KEGG ID	Protein name	Source					
			Here	Ref. ^54^	Ref. ^53^	Ref. ^52^	Ref. ^19^	Ref. ^51^
CysD	PA4443	Sulfate adenylate transferase subunit 2	↑	NR	NR	NR	NR	NR
FptA	PA4221	Pyochelin outer membrane receptor	↑	NR	NR	NR	↑	NR
OprD	PA0958	Porin	↑	NR	o	NR	NR	NR
PolA	PA5493	DNA polymerase	↑	NR	NR	NR	NR	NR
AccB	PA4847	Acetyl-CoA carboxylase biotin carboxyl carrier protein	↓	NR	NR	↑	NR	NR
AcnA	PA1562	Aconitate hydratase	↓	NR	NR	↓	NR	NR
AcnB	PA1787	Aconitate hydratase B	↓	NR	NR	o	NR	NR
AotP	PA0892	Arginine/ornithine ABC transporter ATP-binding protein	↓	NR	NR	↓	NR	NR
CcpA	PA4587	Cytochrome C551 peroxidase	↓	NR	NR	↑	NR	NR
CoaD	PA0363	Phosphopantetheine adenylyltransferase	↓	NR	NR	↑	NR	NR
CycH	PA1483	Cytochrome c-type biogenesis protein	↓	NR	NR	o	NR	NR
DadA1	PA5304	D-amino acid dehydrogenase small subunit	↓	NR	NR	↓	NR	NR
DnaK	PA4761	Chaperone protein	↓	↓	NR	↑	↑	NR
Frr	PA3653	Ribosome recycling factor	↓	NR	NR	↑	NR	↑
HemN	PA1546	Oxygen-independent coproporphyrinogen-III oxidase	↓	NR	NR	NR	NR	NR
IlvD	PA0353	Dihydroxy-acid dehydratase	↓	NR	NR	NR	NR	NR
KatA	PA4236	Catalase	↓	↑	NR	o	↑	NR
MtnB	PA1683	Methylthioribulose-1-phosphate dehydratase	↓	NR	NR	↑	NR	NR
NfuA	PA1847	Fe/S biogenesis protein	↓	NR	NR	↑	NR	NR
NirS	PA0519	Nitrite reductase	↓	NR	NR	↓	NR	NR
NuoB	PA2638	NADH-quinone oxidoreductase subunit B	↓	NR	NR	NR	NR	NR
NuoC	PA2639	NADH-quinone oxidoreductase subunit C/D	↓	NR	NR	NR	NR	NR
NuoE	PA2640	NADH-quinone oxidoreductase subunit E	↓	NR	NR	NR	NR	NR
NuoF	PA2641	NADH dehydrogenase I subunit F	↓	NR	NR	o	NR	NR
NuoG	PA2642	NADH-quinone oxidoreductase subunit G	↓	NR	NR	NR	NR	NR
NuoI	PA2644	NADH-quinone oxidoreductase subunit I	↓	NR	NR	↑	NR	NR
OprF	PA1777	Outer membrane porin F	↓	o	↓	↑	NR	NR
PA1673	PA1673	Bacteriohemerythrin	↓	NR	NR	NR	NR	NR
PA2953	PA2953	Electron transfer oxidoreductase	↓	NR	NR	↓	NR	NR
PA5475	PA5475	Hypothetical protein	↓	NR	NR	NR	NR	NR
PagL	PA4661	Lipid A 3-O-deacylase	↓	NR	NR	o	NR	NR
PckA	PA5192	Phosphoenolpyruvate carboxykinase	↓	NR	NR	NR	NR	↑
PhhA	PA0872	Phenylalanine 4-monooxygenase	↓	NR	NR	NR	NR	NR
PurT	PA3751	Phosphoribosylglycinamide formyltransferase	↓	NR	NR	o	NR	NR
RplL	PA4271	50S ribosomal protein L7/L12	↓	NR	NR	NR	NR	o
RplM	PA4433	50S ribosomal protein L13	↓	NR	NR	↓	NR	NR
RplT	PA2741	50S ribosomal protein L20	↓	NR	NR	↑	NR	NR
RpmD	PA4245	50S ribosomal protein L30	↓	NR	NR	NR	NR	NR
RpmE	PA5049	50S ribosomal protein L31	↓	NR	NR	NR	NR	NR
RpsL	PA4268	30S ribosomal protein S12	↓	NR	NR	↓	NR	NR
SecG	PA4747	Preprotein translocase subunit	↓	NR	NR	NR	NR	NR
TatA	PA5068	Twin-arginine translocation protein	↓	NR	NR	NR	NR	NR

Includes previous data

“o” indicates no significant difference in relative protein abundance in planktonic vs. biofilm cultures

“NR” indicates protein not reported

### Proteomics-based comparison of biofilm vs. planktonic cultures

**Fig. 5:**
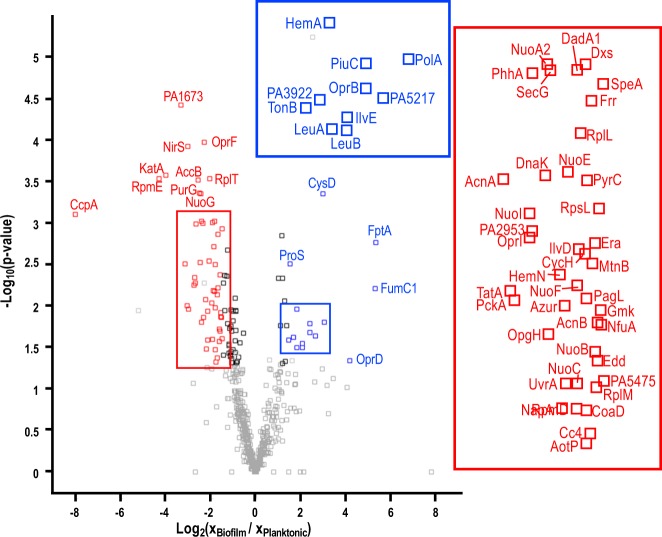
*P. aeruginosa* protein abundances changes for biofilm vs. planktonic cultures grown on LCSP medium for 11 h. Proteins with |log_2_(*x*_Biofilm_/*x*_Planktonic_)| > 1.5 are deemed significantly lower or higher abundance and are annotated as red or blue points, respectively

Protein abundances were also compared for biofilm vs. planktonic cultures, identifying 66 and 105 proteins that had significant changes in abundance for growth on LCSP or CSP medium, respectively, as shown in Fig. 5, Supplementary Fig. 2, and Supplementary Tables 4 and 5. Those proteins that displayed significant changes in abundance for both LCSP and CSP media are tabulated in Table 4. Four proteins displayed higher abundances and 38 proteins displayed lower abundances for biofilm vs. planktonic cultures. These 42 proteins were compared to those previously reported in *P. aeruginosa* biofilm studies, as shown in Table 4.^19,51–54^

The planktonic cultures had higher abundances of key respiration proteins associated with NADH dehydrogenase I and cytochrome C (NuoB, NuoC, NuoE, NuoF, NuoG, NuoI, and CycH) and as well as central metabolism and TCA cycle enzymes (AcnA, AcnB, and PckA), presumably due to higher concentrations of dissolved oxygen. Cellular stress, iron metabolism, and oxidative stress proteins were also at higher abundance during planktonic growth due likely to the presence of oxygen (DnaK, KatA, CcpA, NirS, and NfuA). The planktonic cultures had higher growth rates than the biofilm cultures, as reflected in the proteome with higher abundance of ribosome-associated proteins (RpmD, RpmE, RpsL, RplL, RplM, RplT, and Frr). Cytochrome C551 peroxidase (CcpA) had the highest relative abundance for any identified protein for planktonic growth on both LCSP and CSP media.

Biofilm cultures had higher abundances of pyochelin transporter protein (FptA); sulfur metabolism protein (CysD), involved in the biosynthesis of cysteine, where two cysteines and a salicylate make up the siderophore pyochelin; and porin (OprD). The increased abundance of DNA polymerase (PolA) is consistent with the importance of DNA as a component of *P. aeruginosa* biofilms (see Table 2).^55^ Iron acquisition protein (FptA) had the highest relative abundance of any identified protein for biofilm cultures grown on either LCSP or CSP medium. Additional notable iron metabolism proteins with higher biofilm abundance in only LCSP-grown cultures were glutamyl-tRNA reductase (HemA) and iron siderophore transporter (TonB, see Fig. 5).

## Discussion

CCR is an important driver of growth, cellular energy, and structural maintenance.^56,57^ The common paradigm for CCR describes a preference for glucose over other substrates, including organic acids; this metabolic behavior is known as diauxie.^7,10^ The preference for sugars typically results in the common metabolic strategy known as “overflow” where the preferred carbon source is only partially oxidized and compounds such as acetate are secreted as byproducts in the presence of oxygen.^38–40^ In contrast, *P. aeruginosa* has evolved a preference for organic acids over glucose, which is a regulation scheme known as reverse diauxie.^7,10^ The repression of glucose utilization for the preferred organic acid uptake has been reported in planktonic *Pseudomonads*.^10,58^ While reverse diauxie is generally inferred based on planktonic growth, the authors found no prior reports of explicit documentation of reverse diauxie by *P. aeruginosa* biofilms via exometabolomics and proteomics.^59,60^

Chronic wounds are categorized as nonhealing wounds that do not proceed through resolution within a standard period of time of 2–3 months.^61^ Nonhealing, chronic wounds impact over eight million US patients with annual healthcare costs approaching ~$60 billion (USD).^37,62^ High lactate levels are present in chronic wounds and have been correlated with disease severity,^63,64^ with the highest levels of lactate reported among obese patients with type II diabetes.^65^ Wound healing can be impaired at high lactate concentrations where host cell viability is severely compromised.^66^ It has also been postulated that lactate is required for microbial invasion and replication.^59^ Lactate utilization contributes to pathogenesis and infection processes by enhancing resistance to oxygen-dependent bactericidal mechanisms by competing for oxygen, stimulating microbial growth rates, and providing a more direct pathway for pathogenic determinants, including polysialic acid capsule and sialylated lipopolysaccharide.^67^ Furthermore, the presence of lactate induces mechanisms that may protect microorganisms from antimicrobial agents. Spermidine binds to lipopolysaccharides to stabilize and protect the outer bacterial membrane as a defensive mechanism against antibiotics and oxidative stress.^68^ The addition of lactate to the medium increased the abundance of polyamine aminopropyltransferase 1 (SpeE1), which participates in the synthesis of spermidine. High concentrations of lactate could therefore induce mechanisms that protect bacteria from antimicrobial agents.

*P. aeruginosa* prefers lower energy organic acid substrates, relative to glucose. The substrate preference obligates the presence of a terminal electron acceptor like oxygen for respiratory catabolism. Respiration pathways in turn create an obligate requirement for metal cofactors such as iron and zinc for enzyme function. Metals are often limiting in biofilms due to low solubility in aerobic environments and the necessity of diffusion through the biofilm. Metal limitations can induce synthesis of virulence- associated proteins including L-ornithine N(5)-monooxygenase (PvdA), fumarate hydratase (FumC1), and superoxide dismutase [Mn] (SodA).^69–71^ For instance, PvdA catalyzes the first step in the biosynthesis of pyoverdin siderophores, a virulence factor that is typically induced under iron-starvation conditions along with exotoxin A and endoproteases.^70^ Induction of GTP cyclohydrolase (FolE2) has been found to occur under zinc-deficient conditions.^71^ Figure 4 shows that growth on lactate resulted in higher abundance of these three virulence-associated proteins.

*P. aeruginosa* is a very competitive and therefore widely distributed microorganism found globally in medical, community, aquatic, and terrestrial environments.^72^ The ecological basis of the reverse diauxie strategy, which is contrary to classic CCR, is still open to debate. Common hypotheses are related to the availability of substrates during the organisms’ evolutionary histories. In many environments, such as soils, organic acids are plentiful byproducts of sugar-preferring microbial life. The availability of those organic acids and their inhibitory effects at higher concentrations may have been strong factors for the evolution of reverse diauxie metabolisms. The coexistence of multiple species expressing complementary phenotypes could also explain the presence of a reverse diauxie. Reverse diauxie phenotypes existing in conjunction with classic diauxie phenotypes could result in a potentially competitive resource allocation between cross-feeding populations enhancing productivity and stability.^73,74^

Analysis of biofilms entails complexities not typical to planktonic cultures. Cellular phenotypes during planktonic growth are generally homogenous, whereas phenotypes found in biofilm grown cultures can be heterogeneous due to spatially dependent chemical gradients and nutrient availability.^75,76^ Because there was no direct method to compare relative growth phases between the biofilm and planktonic growth, the same culturing time points were used.

An indeterminate source of error in the proteomics results presented here is the intensity-based quantification method used by the Perseus software, which relies upon the sum of peptide ion intensities to determine relative protein abundances.^77^ This strategy assumes no differences in protein extraction efficiency from the initial sample preparation as well as uniform ionization efficiencies of different peptide ions in electrospray ionization MS. However, these assumptions may not be that consequential compared to the order of magnitude differences in protein abundances measured here.

Some proteins observed in the current study have been reported in previous *P. aeruginosa* biofilm studies (see Table 4),^19,51–54^ although many more of the proteins observed here were previously reported in a prior study of cell adhesion.^24^ The protein trends are not always consistent between the different biofilm studies. For example, chaperone protein (DnaK) has been shown in multiple studies to either increase or decrease in abundance relative to biofilm phenotype (see Table 4).^19,51–54^ Differences in the trends could be a result of strain-specific adaptations, medium-specific acclimations, differences in the culturing phase, a result of the different proteomic technologies (gel vs. gel free), or different omics statistical software.

A CCR strategy known as reverse diauxie was documented here in biofilm cultures of *P. aeruginosa*. Reverse diauxie has been observed previously in planktonic cultures of *Pseudomonads* but, to the authors’ knowledge, has not been directly recorded in biofilm cultures. Biofilm vs. planktonic comparisons for cultures grown on LCSP or CSP medium identified 66 and 105 proteins, respectively, that had significant changes in abundance. Of those, 4 proteins displayed higher abundances and 38 proteins displayed lower abundances for biofilm vs. planktonic growth conditions in both growth media.

This study also quantifies how lactate can affect biofilm phenotype, increasing the abundance of virulence-associated proteins. The key lactate catabolizing enzyme LldD display a higher abundance in LCSP-grown cultures. Chronic wounds often have elevated levels of lactate and often are colonized by microorganisms growing as biofilms, which hinders the healing processes. However, lower abundances were observed for the glucose catabolism-associated proteins such as OprB and Glk as well as the glycolysis pathway enzymes Zwf, Pgl, and Gap. Overall, 50 and 51 proteins from the planktonic and biofilm cultures, respectively, were observed with statistically significant differences in abundance in LCSP vs. CSP media.

Ultimately, the presented data make substantial contributions to the growing *P. aeruginosa* omics databases. Understanding these phenomena will provide new opportunities to manipulate these biofilms, including potential new strategies for treating chronic wound infections. However, it is still a major challenge to systematically interpret the data. The complexity of the proteomic response of this clinical strain of *P. aeruginosa* under the conditions examined here motivate the application of multiple pathway and computational metabolic modeling.^36,74^

## Methods

### Bacterial strain and culturing conditions

Overnight cultures of *P. aeruginosa* chronic wound clinical isolate strain 215^78^ were grown in 10 mL of chemically defined medium (CSP) containing 4 g/L glucose, 0.7 g/L sodium citrate, 0.1 g/L EDTA tetrasodium salt, 1.7 g/L yeast nitrogen base, minimum essential media non-essential amino acid (100× solution), minimum essential media amino acid (50× solution), 4.7 g/L KH_2_PO_4_, 8.2 g/L Na_2_HPO_4_, 0.02 g/L adenine, 0.02 g/L uracil, 0.02 g/L cytosine, 0.02 g/L guanine, 0.15 g/L glutamine, 2.0 × 10^−6^ g/L vitamin B_12_, 2.8 × 10^−3^ g/L FeSO_4_·7H_2_O, and 1.2 × 10^−5^ g/L CoCl_2_·6H_2_O or CSP supplemented with 2 g/L lactate (LCSP). Aliquots of the overnight cultures were diluted to an optical density at 600 nm (OD_600_) of ~0.010 with fresh medium in a baffled shake flask covered with a gas permeable cap and grown in a shaker incubator under aerobic conditions at 37 °C and 150 oscillations/min. For biofilm growth, 100 µL of diluted overnight cultures (~10^7^ colony-forming units/mL) were used to inoculate tissue culture inserts suspended in 6-well plates above fresh CSP medium and grown in a shaker incubator under aerobic conditions at 37 °C and 150 oscillations/min. This biofilm culturing method was modified from Ammons et al.^37^ Three separate biofilms were each viewed and imaged one time on each of 6 days using OCT imaging at a nominal wavelength of 930 nm (model GAN 210 Ganymed Microscope with an LSM03 probe, KEYENCE Corp of America, Itasca, IL). Biofilm cell counts were determined by sampling cultures destructively at the designated time points. Biofilms, attached to membranes, were cut under sterile conditions from the tissue culture inserts, placed in 5 mL of phosphate-buffered saline (PBS) where the biofilms and membranes were separated, then the membranes were removed and discarded. Biofilm cells were disaggregated using a tissue homogenizer, samples were diluted in PBS, and OD_600_ were measured. Biofilm cell counts were determined using an experimentally measured OD_600_ to cell count calibration curve, as shown in Supplementary Fig. 1.

### Exometabolomic analysis

Exometabolomic samples were analyzed using either HPLC or NMR. Supernatants were collected from *P. aeruginosa* planktonic and biofilm cultures. For HPLC extracellular metabolite analyses, 1.5 mL of culture supernatant was filtered using a 0.22-μm syringe filter, mixed with fucose as internal standard, and separated through LC (1200 series, Agilent, Santa Clara, CA) equipped with both a variable wavelength detector and refractive index detector with an ion exclusion column (Aminex HPX-87H, 9 μm particle size, 300 mm length × 7.8 mm internal diameter, Bio-Rad, Hercules, CA) using a 0.03% H_2_SO_4_ isocratic mobile phase, at 0.6 mL/min, for a total run time of 25 min at 45 °C to detect glucose and organic acids. HPLC results are the average of two replicate measurements for each of the three distinct cultures made for each condition (CSP or LCSP) at each noted sampling time for planktonic and biofilm cultures, respectively, with the standard deviation given as the error.

For NMR analysis, supernatants from planktonic cultures were collected by spinning at 4400 × *g* for 5 min. Supernatants from the biofilm samples were collected from the tissue culture wells and filtered through 0.2-μm nylon syringe filters. All supernatant samples were stored at −20 °C until NMR analysis. Proton (^1^H) NMR spectra were acquired at 298 K on a 600-MHz solution NMR spectrometer (AVANCE III equipped with a SampleJet automatic sample loading system, Bruker) with 5 mm triple resonance (^1^H, ^15^N, ^13^C) liquid helium-cooled cryoprobe (TCI), and Topspin software (version 3.6.0).^37^ Pulse sequence settings were based on standard recommendations by the Chenomx NMR software suite (version 7.6, Chenomx, Edmonton, Alberta, Canada). NMR spectra were individually baseline-corrected for each sample, with additional spectral processing and analysis performed using the Chenomx software. The Chenomx small-molecule library for 1D ^1^H, 600 MHz NMR was used for metabolite identification and spectral patterns were manually fitted for each sample independently. An internal standard of 4,4-dimethyl-4-silapentane-1-sulfonic acid was used for quantitation of identified metabolites. NMR data for the 9-h planktonic culture is the average of a single measurement of each of three cultures, with an average error of <10 % of the mean value. NMR data for other time points represent a single measurement of a single biological sample and is shown only to indicate the expected trends.

Oxygen concentration profiles within the biofilm were measured using a system that precisely positions an oxygen microsensor. This micropositioning system consists of a 25-µm electrochemical oxygen microsensor held by a motorized and computer-controlled micromanipulator and microscope (OX-25 microsensor, MM33-2 MicroProfiling System, and SensorTrace Logger software from Unisense, Aarhus, Denmark). The microsensor was calibrated with a strong reductant solution with both ascorbic acid and sodium hydroxide at a final concentration of 0.1 M and water fully air saturated with vigorous bubbling for 5 min. The oxygen-selective electrode was positioned with the micromanipulator on the biofilm sample using a microscope. Oxygen gradients were measured for two biofilms at every 25 µm from the top of the biofilm and the averages at each point reported.

### Sample preparation for proteomics

Planktonic *P. aeruginosa* and biofilms were transferred into a 2-mL microcentrifuge vial with a screw cap that allowed for mechanical bead beating. Planktonic cells were centrifuged at 3600 × *g* with three PBS wash steps to remove residual media. No wash step was performed for biofilms. Planktonic and biofilm cultures were re-suspended in 1.25 mL radioimmunoprecipitation assay buffer (50 mM Tris-HCl, pH 8.0, 150 mM sodium chloride, 1.0% Igepal CA-630 (NP-40), 0.5% sodium deoxycholate 0.1% sodium dodecyl sulfate) with 12.5 μL of protease inhibitors (Halt Protease Cocktail Inhibitor, Thermo Fisher Scientific, Rockford, IL) for the prevention of enzyme degradation upon cell lysis, 0.1 mg/mL lysozyme for solubilization of the peptidoglycan layer, and 5 mM dithiothreitol (DTT) for disulfide bond cleavage. The remainder of the vial was filled with 0.1 mm diameter zirconia/silica beads. Cells were mechanically lysed at 4800 oscillations/min in a mini bead beater (Mini-beadbeater-1, BioSpec Products, Inc, Bartlesville, OK) for five 30-s cycles with ice water bath chilling in between cycles.

A detergent compatible protein assay kit (DC Bradford Reagent, Thermo Fisher Scientific) was used to determine protein concentrations. An equal amount of protein was transferred to centrifugal filter units (Microcon-30kDa Centrifugal Filter Unit with Ultracel-30 membrane, Millipore Sigma, Billerica, MA) for sample processing following the protocol for filter-aided sample preparation (http://www.biochem.mpg.de/226356/FASP). Briefly, the steps involved buffer exchange with urea for detergent removal, DTT for protein reduction of disulfide bonds, iodoacetamide for alkylation preventing reformation of disulfide bonds, and overnight trypsin digestion at 37 °C with 1:50 enzyme:substrate (w:w) ratio for proteolytic cleavage. The tryptic digests were acidified with formic acid, desalted using a C18 column (Macro SpinColumn, Harvard Apparatus, Holliston, MA), and dried through centrifugal evaporation. Prior to analyses, peptides were re-suspended in 5% acetonitrile with 0.1% formic acid.

### Liquid chromatography tandem mass spectrometry

Planktonic (*N*_(L)CSP_ = 3, single replicate injection) and biofilm (*N*_(L)CSP_ = 2, triple replicate injections) peptide resuspensions were separated through HPLC (1260 Infinity LC System, Agilent, Santa Clara, CA) with a C18 column (3.5 μm particle size, 150 mm length × 75 μm internal diameter, Zorbax 300SB, Agilent) and a 60-min increasing organic gradient from 5% to 85% (mobile phase A: 0.1% formic acid in water and mobile phase B: 0.1% formic acid in acetonitrile) for a total run time of 75 min at a flow rate of 250 nL/min. Following chromatographic separation, peptides were simultaneously ionized in the electrospray ionization source at 1.90 kV spray voltage and 275 °C capillary temperature. Peptide ions were analyzed in a high mass resolution, tandem mass spectrometer (Orbitrap Velos Pro, Thermo Fisher Scientific, Waltham, MA) with automatic gain control at 10^6^ ions and between 1 and 200 ms injection time. Full-scan mass spectra were collected from *m*/*z* 400–2000 in a data-dependent acquisition mode at 30,000 mass resolution. From each full mass spectrum, ten precursor ions were selected for MS/MS analyses in high energy collision-induced dissociation mode using 30% energy for fragmentation.

### Qualitative and quantitative proteomics

Raw mass spectra data files were directly uploaded and processed for protein identification and quantitation using the MaxQuant software (v. 1.5.3.30).^79^ A FASTA file of reviewed *P. aeruginosa* proteins used for MaxQuant identification was downloaded from the UniProt Knowledgebase.^80^ The main search parameters for quantitation were set at 4.5 ppm peptide tolerance, 20 ppm MS/MS match tolerance, 10 ppm MS/MS de novo tolerance, 7 min peptide length, 0.01 False Discovery Rate, carbamidomethyl fixed modification, oxidation and acetylation variable modifications, and enabled contaminant search.

Protein abundances were evaluated by Student’s sample *t* test with *p* value < 0.05 using the Perseus software (v.1.5.4.0)^77^ for statistical significance. Intensity-based quantification by the MaxQuant software^79^ was used to determine relative protein abundances by summing all unique peptide ion intensities from the full LC-MS/MS scans. Perseus software^77^ was used for further data processing where ion intensities were converted into logarithmic scale (base 2) for normalization, then Student’s paired *t* test was applied to obtain *p* values to distinguish statistically significant proteins and log_2_ changes in protein abundances between two culturing conditions. Proteins were only deemed statistically significant when they displayed a *p* value < 0.05. The result of this analysis for all significant protein abundance changes were displayed as “Volcano” or scatter plots of the −log_10_(*p* values) for statistical significance vs. log_2_(*x*_LCSP_/*x*_CSP_), the latter calculated from Student’s *t* test where *x*_(L)CSP_ is a given peptide ion intensity from LC-MS/MS data from the LCSP or CSP culturing condition. For a comparison of planktonic vs. biofilms grown in (L)CSP medium, Welch’s *t* test was used to distinguish statistically significant proteins with *p* value < 0.05 for unequal sample sizes. Results were again displayed as “Volcano” plots, highlighting statistical significance, −log_10_(*p* values), vs. protein abundance change, log_2_(*x*_Biofilm_/*x*_Planktonic_).

Protein abundance changes were deemed significant when |log_2_(*x*_LCSP_/*x*_CSP_)| > 1.5 or |log_2_(*x*_Biofilm_/*x*_Planktonic_)| > 1.5 and either a minimum number of two unique peptides or sequence coverage >10% were functionally annotated using The Search Tool for Retrieval of Interacting Genes database (STRING, v. 10.5)^81^ for protein–protein interactions (set at medium confidence of 0.4) as well as the Pseudomonas Genome Database^44^ and the UniProt Knowledgebase.^80^ For lactate and glucose metabolic pathways, the KEGG database was also used for ref. ^60^

### Reporting summary

Further information on research design is available in the Nature Research Reporting Summary linked to this article.

## Supplementary information

Reporting Summary

Supplemental Material

## Data Availability

The proteomic data that support the findings of this study are available on the Mass Spectrometry Interactive Virtual Environment (MassIVE) repository at ftp://massive.ucsd.edu/MSV000084204/. The remaining data that support the findings of this study are available from the corresponding author upon reasonable request.
