# Supplementary material for: Reverse diauxie phenotype in Pseudomonas aeruginosa biofilm revealed by exometabolomics and label-free proteomics
Source: NPJ Biofilms Microbiomes. 2019 Oct 25;5:31. doi: 10.1038/s41522-019-0104-7 (PMC6814747; doi:10.1038/s41522-019-0104-7)
Supplement: Supplementary file 2 — Supplemental Material [file 41522_2019_104_MOESM2_ESM.pdf]

Supplementary Information for

Reverse Diauxie Phenotype in *Pseudomonas aeruginosa* Biofilm Revealed by Exometabolomics and Label-Free Proteomics

Yeni P. Yung,[1] S. Lee McGill,[2] Hui Chen,[3] Heejoon Park,[2] Ross P. Carlson,[2] and Luke Hanley[1,*]

[1]Department of Chemistry, University of Illinois at Chicago
[2]Center for Biofilm Engineering, Montana State University
[3]Research Resources Center, University of Illinois at Chicago

See Methods in primary manuscript for sample sizes, replicates, and error analysis for data reported here.

Supplementary Table 1. NMR exometabolomics data for biofilm and planktonic *P. aeruginosa* cultures grown on CSP medium. <0.1 mM indicates metabolite was detected at concentrations below this level.

Biofilm data					Planktonic data			
**Time (h)**	**0**	**6**	**12**	**18**	**Time (h)**	**9**	**Time (h)**	**13**
**Compound**	**mM**	**mM**	**mM**	**mM**	**Compound**	**mM**	**Compound**	**mM**
Adenine	0.2	0.3	0.1	<0.1	2-Hydroxybutyrate	<0.1	Histidine	<0.1
Alanine	1.0	1.1	0.3	0.1	Acetate	<0.1	Anthranilate	<0.1
Arginine	1.4	1.9	0.9	0.3	Adenine	<0.1	Ethanol	0.1
Asparagine	0.8	0.9	<0.1	<0.1	Alanine	0.6 ± 0.1	DSS	0.1
Aspartate	1.2	1.1	0.3	0.1	Arginine	1.6 ± 0.2	Epicatechin	0.2
Cadaverine	<0.1	<0.1	<0.1	0.2	Asparagine	0.3 ± 0.2		
Citrate	2.6	5.2	1.2	1.1	Aspartate	0.2 ± 0.1		
Cystine	0.6	0.9	0.6	0.3	Cellobiose	0.1 ± 0.05		
Cytosine	0.2	0.3	0.1	0.1	Citrate	3.4 ± 0.05		
DSS	0.3	0.2	0.2	0.2	Cysteine	<0.1		
Ethanol	<0.1	0.1	0.1	0.1	Cystine	0.5 ± 0.05		
Gluconate	<0.1	<0.1	0.3	0.4	Cytosine	0.1 ± 0.05		
Glucose	24.6	31.5	20.0	10.5	DSS	0.3 ± 0.05		
Glutamate	1.0	1.3	0.4	0.2	Dimethylamine	<0.1		
Glycine	1.1	1.4	0.4	0.1	Ethanol	0.1 ± 0.05		
Histidine	0.5	0.7	0.3	0.1	Formate	<0.1		
Isoleucine	1.0	1.4	0.5	0.2	Fumarate	<0.1		
Leucine	0.9	1.1	0.5	0.2	Glucose	27.5 ± 4.5		
Lysine	1.0	1.4	0.6	<0.1	Glutamate	0.5 ± 0.05		
Methionine	0.3	0.4	0.1	0.1	Glutamine	<0.1		
Phenylalanine	0.5	0.6	0.2	0.1	Glycine	1.0 ± 0.05		
Proline	1.1	1.3	0.7	<0.1	Histidine	0.6 ± 0.05		
Serine	1.8	2.6	0.9	0.3	Hypoxanthine	<0.1		
Threonine	1.4	1.4	0.6	0.3	Isoleucine	1.0 ± 0.05		
Tryptophan	0.1	0.2	0.1	<0.1	Isopropanol	<0.1		
Tyrosine	0.6	0.8	0.3	0.1	Lactate	<0.1		
Uracil	0.2	0.3	0.8	<0.1	Leucine	1.3 ± 0.1		
Valine	1.0	1.5	0.5	0.3	Lysine	0.9 ± 0.1		

Methionine	0.2 ± 0.05
Phenylalanine	0.4 ± 0.05
Proline	0.7 ± 0.05
Pyroglutamate	0.1 ± 0.05
Serine	0.8 ± 0.05
Succinate	<0.1
Tartrate	<0.1
Threonine	1.3 ± 0.1
Tryptophan	0.1 ± 0.05
Tyrosine	0.7 ± 0.05
Uracil	0.1 ± 0.05
Valine	1.0 ± 0.1
Xanthine	<0.1

Supplementary Table 2. Inclusive list of metabolically relevant, *P. aeruginosa* proteins with significant increases (blue highlighted, ↑), significant decreases (red highlighted, ↓), and non-significant abundance changes (no highlights) during planktonic growth in LCSP vs. CSP media (see Figure 4a). Proteins in boldface are observed in both planktonic (Figure 4a) and biofilm (Figure 4b) cultures regardless of abundance. '*' indicates homologous protein KEGG ID.

Protein	KEGG ID	Description of Proteins in Figure 4a	Unique Peptides	Sequence Coverage %	$\log_2(x_{LCSP}/x_{CSP})$
**LldD**	**PA4771**	**L-lactate dehydrogenase**	**14**	**44.4**	**4.88 ↑**
MmsA	PA3570	methylmalonate-semialdehyde dehydrogenase	18	50.1	-5.47 ↓
MmsB	PA3569	3-hydroxyisobutyrate dehydrogenase	9	52.7	-4.53 ↓
HutH	PA5098	histidine ammonia-lyase	8	27.5	-4.02 ↓
Glk	PA3193	glucokinase	2	12.1	-3.82 ↓
**OprB**	**PA3186**	**porin B**	**15**	**44.3**	**-2.98 ↓**
HutU	PA5100	urocanase	23	55.3	-2.89 ↓
Zwf	PA3183	glucose-6-phosphate 1-dehydrogenase	18	53.0	-2.30 ↓
HupA	PA5348	DNA-binding protein	9	73.3	-2.30 ↓
AtoB	PA2001	acetyl-CoA acetyltransferase	15	62.6	-2.28 ↓
Gap	PA3195	glyceraldehyde 3-phosphate dehydrogenase	16	59.0	-2.08 ↓
MaiA	PA2007	maleylacetoacetate isomerase	5	39.2	-1.99 ↓
**HutI**	**PA5092**	**imidazolonepropionase**	**3**	**11.2**	**-1.78 ↓**
Pgl	PA3182	6-phosphogluconolactonase	9	52.1	-1.51 ↓
Hfq	PA4944*	RNA-binding protein	16	51.3	-1.49 ↓
Eda	PA3181	2-dehydro-3-deoxy-phosphogluconate aldolase	8	51.4	-1.44
GlyA2	PA2444	serine hydroxymethyltransferase	5	27.0	-1.35
PhhC	PA0870	aromatic amino acid aminotransferase	18	69.2	-1.18
AstE	PA0901	succinylglutamate desuccinylase	3	16.0	-1.05
AspA	PA5429	aspartate ammonia-lyase	17	48.7	-1.04
SpuD	PA0300	putrescine ABC transporter substrate-binding protein	12	52.6	-0.99
BraF	PA1071	ABC transporter ATP-binding protein	3	18.0	-0.94
GdhB	PA3068	NAD-specific glutamate dehydrogenase	58	48.1	-0.88
PhhB	PA0871	pterin-4-alpha-carbinolamine dehydratase	7	78.8	-0.88
BraG	PA1070	ABC transporter ATP-binding protein	5	29.2	-0.87
DavT	PA0266	5-aminovalerate aminotransferase	18	72.8	-0.79
TyrS	PA4138*	tyrosyl-tRNA synthetase	1	55.3	-0.57
GltA	PA1580	citrate synthase	21	76.6	-0.55
ProS	PA0956*	prolyl-tRNA synthetase	2	58.0	-0.53
SahH	PA0432	adenosylhomocysteinase	24	68.7	-0.40
NfuA	PA1847	Fe/S biogenesis protein	5	46.9	-0.40
AhpC	PA0139*	alkyl hydroperoxide reductase subunit C	13	78.6	-0.39
PA5475	PA5475	hypothetical protein	8	73.1	-0.39
PA1673	PA1673	bacteriohemerythrin	6	50.3	-0.33
Hom	PA3736	homoserine dehydrogenase	10	38.0	-0.27
ArgB	PA5323	acetylglutamate kinase	11	15.4	-0.24
MexA	PA0425	multidrug resistance protein	14	57.2	-0.22
RnpA	PA5569	ribonuclease P	1	11.9	-0.20

Protein	KEGG ID	Description of Proteins in Figure 4a	Unique Peptides	Sequence Coverage %	$\log_2(x_{LCSP}/x_{CSP})$
**CysS**	**PA1795**	**cysteine--tRNA ligase**	**12**	**32.4**	**-0.17**
LpdG	PA1587	2-oxoglutarate dehydrogenase complex dihydrolipoyl dehydrogenase	23	57.9	-0.13
KatA	PA4236	catalase	9	36.1	-0.07
Pta	PA0835	phosphate acetyltransferase	9	21.2	-0.06
AlgU	PA0762	RNA polymerase sigma factor	3	31.1	0.14
RplV	PA4258	50S ribosomal protein L22	9	54.5	0.15
ArgG	PA3525	argininosuccinate synthase	19	64.9	0.16
**PckA**	**PA5192***	**phosphoenolpyruvate carboxykinase (ATP)**	**2**	**61.8**	**0.24**
RplP	PA4256	50S ribosomal protein L16	7	48.2	0.28
**SpeE1**	**PA1687**	**polyamine aminopropyltransferase**	**8**	**43.7**	**0.48**
**DsbA**	**PA5489**	**thiol:disulfide interchange protein**	**8**	**44.5**	**0.49**
RapA	PA3308	RNA polymerase-associated protein	16	24.3	0.76

Supplementary Table 3. Inclusive list of metabolically relevant, *P. aeruginosa* proteins with significant increases (blue highlighted, ↑), significant decreases (red highlighted, ↓), and non-significant abundance changes (no highlights) during biofilm growth in LCSP vs. CSP media (see Figure 4b). Proteins in boldface are observed in both planktonic (Figure 4a) and biofilm (Figure 4b) cultures, regardless of abundance. '*' indicates homologous protein KEGG ID.

Protein	KEGG ID	Description of Proteins in Figure 4b	Unique Peptides	Sequence Coverage %	$\log_2(x_{LCSP}/x_{CSP})$
FumC1	PA4470	fumarate hydratase	19	59.6	1.51 ↑
PA5217	PA5217	iron ABC transporter substrate-binding protein	12	36.1	1.61 ↑
HisA	PA5141	1-(5-phosphoribosyl)-5-[(5-phosphoribosylamino)methylideneamino] imidazole-4-carboxamide isomerase	6	32.7	1.61 ↑
SodA	PA4468	superoxide dismutase	3	25.1	1.78 ↑
Mqo1	PA3452	malate:quinone oxidoreductase	4	11.1	1.87 ↑
**SpeE1**	**PA1687**	**polyamine aminopropyltransferase**	**3**	**23.8**	**2.04** ↑
PvdA	PA2386	L-ornithine N5-oxygenase	4	12.4	2.10 ↑
FolE2	PA5539	GTP cyclohydrolase	6	36.2	2.92 ↑
**LldD**	**PA4771**	**L-lactate dehydrogenase**	**12**	**39.6**	**5.26** ↑
**HutI**	**PA5092**	**imidazolone-5-propionate hydrolase**	**4**	**16.2**	**-1.37**
**OprB**	**PA3186**	**porin B**	**14**	**41.0**	**-0.85**
LeuC	PA3121	3-isopropylmalate dehydratase large subunit	4	14.3	-0.67
TrpS	PA4439	tryptophan--tRNA ligase	10	37.7	0.44
AtpA	PA5556*	ATP synthase F1, alpha subunit	1	39.7	0.63
NqrC	PA2997	Na(+)-translocating NADH-quinone reductase subunit C	4	23.4	0.64
CspA	PA3266	major cold shock protein	3	52.2	0.69
**PckA**	**PA5192***	**phosphoenolpyruvate carboxykinase (ATP)**	**1**	**53.8**	**0.76**
Hcp1	PA0085	protein secretion apparatus assembly protein	6	38.9	0.78
SecD	PA3821	preprotein translocase subunit	12	23.4	0.79
RdgC	PA3263	putative recombination associated protein	13	64.4	0.81
MurG	PA4412	undecaprenyldiphospho-muramoylpentapeptide beta-N-acetylglucosaminyltransferase	4	23.0	0.82
**CysS**	**PA1795**	**cysteine--tRNA ligase**	**8**	**21.7**	**0.82**
HmuV	PA4706	putative ATP-binding component of ABC transporter	3	25.9	0.84
FtsA	PA4408	cell division protein	14	48.7	0.84
HemC	PA5260*	porphobilinogen deaminase	4	18.8	0.84
ClpP1	PA1801	ATP-dependent Clp protease proteolytic subunit	4	21.1	0.86
PyrH	PA3654	uridylate kinase	8	46.1	0.89
**DsbA**	**PA5489**	**thiol:disulfide interchange protein**	**6**	**28.9**	**0.90**
TrpE	PA0609	anthranilate synthase component I	14	38.8	0.91
Eco	PA2755	ecotin precursor	7	52.6	0.92
SthA	PA2991	soluble pyridine nucleotide transhydrogenase	12	43.1	0.92
ComL	PA4545	competence protein	9	31.1	0.93
TyrS	PA4138	tyrosyl-tRNA synthetase	5	24.5	0.94
LepB	PA0768	signal peptidase I	5	20.8	0.94
TrpC	PA0651	indole-3-glycerol-phosphate synthase	8	30.2	0.96

Protein	KEGG ID	Description of Proteins in Figure 4b	Unique Peptides	Sequence Coverage %	$\log_2(x_{LCSP}/x_{CSP})$
Xpt	PA5298	xanthine phosphoribosyltransferase	2	18.4	0.97
RplL	PA4271	50S ribosomal protein L7/L12	8	86.1	0.98
SecB	PA5128	preprotein translocase subunit	6	58.3	0.99
AceA	PA2634	isocitrate lyase	18	46.7	1.01
HchA	PA1135	molecular chaperone Hsp31/glyoxalase	1	11.7	1.13
ClpV1	PA0090	secretion protein	6	9.3	1.14
PstS	PA5369*	putative phosphate ABC transporter, periplasmic phosphate-binding protein	4	24.1	1.20
PasP	PA0423	hypothetical protein	15	69.6	1.21
ThrB	PA5495	homoserine kinase	2	12.0	1.23
TrmB	PA0382	tRNA (guanine-N(7)-)-methyltransferase	2	9.4	1.23
ArgF	PA3537	ornithine carbamoyltransferase	4	21.6	1.27
PurC	PA1013*	phosphoribosylaminoimidazole-succinocarboxamide synthase	1	34.3	1.32
ThiI	PA5118	thiamine biosynthesis protein	2	12.8	1.35
DnaE	PA3640	DNA polymerase III subunit alpha	6	8.5	1.39
PA1295	PA1295	hypothetical protein	1	24.7	1.39
PA2271	PA2271	acetyltransferase	1	11.7	1.41

Supplementary Table 4. Inclusive list of metabolically relevant, *P. aeruginosa* proteins with significant increases (blue highlighted, ↑), significant decreases (red highlighted, ↓), and non-significant abundance changes (no highlights) during growth in LCSP medium for biofilm vs. planktonic cultures (see Figure 5). Boldface proteins are common to both LCSP (Figure 5) and CSP (Supplementary Figure 2) media, regardless of abundance. '*' indicates homologous protein KEGG ID.

Protein	KEGG ID	Description of Proteins in Figure 5	Unique Peptides	Sequence Coverage %	$\log_2(x_{BF}/x_{Plankt})$
TonB	PA5531	transporter	2	12.0	1.46 ↑
ProS	PA0956*	prolyl-tRNA synthetase	2	44.5	1.53 ↑
PA3922	PA3922	hypothetical protein	16	55.4	1.68 ↑
HemA	PA4666	glutamyl-tRNA reductase	3	15.9	1.83 ↑
LeuA	PA3792*	2-isopropylmalate synthase	1	59.5	1.84 ↑
LeuB	PA3118	3-isopropylmalate dehydrogenase	13	50.8	2.08 ↑
IlvE	PA5013	branched-chain amino acid aminotransferase	9	45.9	2.10 ↑
OprB	PA3186	porin B	12	32.6	2.41 ↑
PiuC	PA4515	hydroxylase	3	16.8	2.42 ↑
PA5217	PA5217	iron ABC transporter substrate-binding protein	13	46.4	2.67 ↑
**CysD**	**PA4443**	**sulfate adenylyltransferase subunit 2**	**6**	**25.9**	**2.99** ↑
**PolA**	**PA5493**	**DNA polymerase I**	**9**	**14.9**	**3.07** ↑
**OprD**	**PA0958**	**porin D**	**12**	**37.5**	**4.19** ↑
**FumC1**	**PA4470**	**fumarate hydratase**	**18**	**58.5**	**5.32** ↑
**FptA**	**PA4221**	**Fe(III)-pyochelin outer membrane receptor**	**40**	**74.9**	**5.33** ↑
**CcpA**	**PA4587**	**cytochrome C551 peroxidase**	**11**	**43.4**	**-7.99** ↓
**RpmE**	**PA5049**	**50S ribosomal protein L31**	**4**	**74.6**	**-4.28** ↓
**KatA**	**PA4236**	**catalase**	**6**	**25.3**	**-3.97** ↓
**PA1673**	**PA1673**	**bacteriohemerythrin**	**6**	**50.3**	**-3.30** ↓
**AcnA**	**PA1562**	**aconitate hydratase**	**30**	**50.9**	**-3.13** ↓
**TatA**	**PA5068**	**twin-arginine translocation protein**	**4**	**63.4**	**-3.03** ↓
**NirS**	**PA0519**	**nitrite reductase**	**13**	**30.1**	**-2.99** ↓
**PckA**	**PA5192***	**phosphoenolpyruvate carboxykinase**	**1**	**59.3**	**-2.97** ↓
**OprI**	**PA2853**	**outer membrane lipoprotein**	**6**	**45.8**	**-2.71** ↓
**NuoI**	**PA2644**	**NADH-quinone oxidoreductase subunit I**	**3**	**17.0**	**-2.70** ↓
**PA2953**	**PA2953**	**electron transfer flavoprotein-ubiquinone oxidoreductase**	**10**	**27.9**	**-2.65** ↓
**PhhA**	**PA0872**	**phenylalanine 4-monooxygenase**	**3**	**13.7**	**-2.64** ↓
**AccB**	**PA4847**	**acetyl-CoA carboxylase biotin carboxyl carrier protein subunit**	**5**	**60.9**	**-2.55** ↓
**PurT**	**PA3751**	**phosphoribosylglycinamide formyltransferase**	**4**	**15.3**	**-2.48** ↓
**DnaK**	**PA4761***	**chaperone protein**	**4**	**19.9**	**-2.44** ↓
**NuoG**	**PA2642**	**NADH-quinone oxidoreductase subunit G**	**21**	**32.5**	**-2.42** ↓
NuoA2	PA2637	NADH-quinone oxidoreductase subunit A	1	12.4	-2.40 ↓
OpgH	PA5077	glucosyltransferase	5	12.2	-2.40 ↓
**SecG**	**PA4747**	**preprotein translocase subunit**	**2**	**37.2**	**-2.34** ↓
**RpmD**	**PA4245**	**50S ribosomal protein L30**	**1**	**24.1**	**-2.31** ↓
**OprF**	**PA1777**	**outer membrane porin F**	**16**	**49.7**	**-2.27** ↓

Protein	KEGG ID	Description of Proteins in Figure 5	Unique Peptides	Sequence Coverage %	$\log_2(x_{BF}/x_{Plankt})$
**HemN**	**PA1546**	**oxygen-independent coproporphyrinogen-III oxidase**	**9**	**32.4**	**-2.20** ↓
NapA	PA1174	nitrate reductase catalytic subunit	5	9.4	-2.17 ↓
Azur	PA4922*	Azurin	1	71.9	-2.13 ↓
UvrA	PA4234	excinuclease ABC subunit A	7	13.2	-2.11 ↓
**NuoE**	**PA2640**	**NADH-quinone oxidoreductase subunit E**	**3**	**25.9**	**-2.06** ↓
**RplT**	**PA2741**	**50S ribosomal protein L20**	**4**	**28.8**	**-2.03** ↓
**NuoF**	**PA2641**	**NADH dehydrogenase I subunit F**	**12**	**40.4**	**-1.93** ↓
**DadA1**	**PA5304**	**D-amino acid dehydrogenase small subunit**	**3**	**8.8**	**-1.90** ↓
**NuoC**	**PA2639**	**NADH:-quinone oxidoreductase subunit C/D**	**13**	**33.1**	**-1.90** ↓
**IlvD**	**PA0353**	**dihydroxy-acid dehydratase**	**9**	**24.8**	**-1.88** ↓
**RplL**	**PA4271**	**50S ribosomal protein L7/L12**	**8**	**86.1**	**-1.85** ↓
**CycH**	**PA1483**	**cytochrome c-type biogenesis protein**	**4**	**19.9**	**-1.80** ↓
**Dxs**	**PA4044**	**1-deoxy-D-xylulose-5-phosphate synthase**	**5**	**12.8**	**-1.80** ↓
**PagL**	**PA4661**	**lipid A 3-O-deacylase**	**5**	**39.9**	**-1.78** ↓
**PyrC**	**PA3527**	**dihydroorotase**	**3**	**18.4**	**-1.77** ↓
**CoaD**	**PA0363**	**phosphopantetheine adenylyltransferase**	**6**	**49.7**	**-1.77** ↓
**AotP**	**PA0892**	**arginine/ornithine ABC transporter ATP-binding protein**	**3**	**20.9**	**-1.76** ↓
**Cc4**	**PA5490**	**cytochrome C4**	**4**	**33.8**	**-1.70** ↓
**MtnB**	**PA1683**	**methylthioribulose-1-phosphate dehydratase**	**3**	**24.9**	**-1.68** ↓
**Frr**	**PA3653***	**ribosome recycling factor**	**1**	**10.8**	**-1.67** ↓
Era	PA0771	GTPase	3	15.4	-1.65 ↓
**RplM**	**PA4433**	**50S ribosomal protein L13**	**6**	**54.9**	**-1.61** ↓
**NuoB**	**PA2638**	**NADH-quinone oxidoreductase subunit B**	**4**	**21.8**	**-1.61** ↓
Edd	PA3194	phosphogluconate dehydratase	12	32.1	-1.58 ↓
**RpsL**	**PA4268**	**30S ribosomal protein S12**	**3**	**25.2**	**-1.56** ↓
**AcnB**	**PA1787**	**aconitate hydratase B**	**40**	**69.5**	**-1.55** ↓
Gmk	PA5336	guanylate kinase	3	18.7	-1.52 ↓
**NfuA**	**PA1847**	**Fe/S biogenesis protein**	**5**	**36.1**	**-1.51** ↓
SpeA	PA4839	arginine decarboxylase	4	8.8	-1.48 ↓
**PA5475**	**PA5475**	**hypothetical protein**	**8**	**55.9**	**-1.48** ↓
**MutS**	**PA3620**	**DNA mismatch repair protein**	**5**	**8.7**	**-1.41**
**GlmU**	**PA5552**	**bifunctional glucosamine-1-phosphate acetyltransferase/N-acetylglucosamine-1-phosphate uridyltransferase**	**6**	**24.2**	**-1.40**
**PA2652**	**PA2652**	**methyl-accepting chemotaxis protein**	**5**	**16.0**	**-1.37**
**RpmB**	**PA5316**	**50S ribosomal protein L28**	**5**	**52.6**	**-1.34**
**PmpR**	**PA0964**	**transcriptional regulator**	**3**	**23.0**	**-1.26**
**RplB**	**PA4260**	**50S ribosomal protein L2**	**13**	**53.1**	**-1.24**
**Ssb**	**PA4232**	**single-stranded DNA-binding protein**	**5**	**30.9**	**-1.23**
**RpsS**	**PA4259**	**30S ribosomal protein S19**	**6**	**49.5**	**-1.19**
**HemB**	**PA5243***	**delta-aminolevulinic acid dehydratase**	**8**	**40.4**	**-1.18**
**DapD**	**PA3666***	**tetrahydrodipicolinate succinylase**	**1**	**39.2**	**-1.17**
**KdsD**	**PA4457**	**arabinose-5-phosphate isomerase**	**5**	**30.7**	**-1.15**

Protein	KEGG ID	Description of Proteins in Figure 5	Unique Peptides	Sequence Coverage %	$\log_2(x_{BF}/x_{Plankt})$
**SodB**	**PA4366**	**superoxide dismutase**	**4**	**37.8**	**-1.12**
**PheS**	**PA2740**	**phenylalanine--tRNA ligase subunit alpha**	**12**	**45.3**	**-1.11**
**RplV**	**PA4258**	**50S ribosomal protein L22**	**9**	**54.5**	**-1.09**
RpmH	PA5570	50S ribosomal protein L34	2	20.5	-1.09
**RplR**	**PA4247**	**50S ribosomal protein L18**	**9**	**58.6**	**-1.08**
**RpsK**	**PA4240**	**30S ribosomal protein S11**	**7**	**60.5**	**-1.08**
**RpsM**	**PA4241**	**30S ribosomal protein S13**	**8**	**70.3**	**-1.05**
**ArcC**	**PA5173**	**carbamate kinase**	**13**	**45.8**	**-1.05**
AlgU	PA0762	RNA polymerase sigma factor	3	42.0	-1.05
**SecY**	**PA4243**	**preprotein translocase subunit**	**6**	**14.7**	**-0.99**
**NagZ**	**PA3005**	**beta-hexosaminidase**	**2**	**11.7**	**-0.98**
UvrB	PA3138	excinuclease ABC subunit B	3	4.9	-0.97
**Mqo1**	**PA3452**	**malate:quinone oxidoreductase**	**7**	**18.2**	**-0.90**
**RplJ**	**PA4272**	**50S ribosomal protein L10**	**7**	**44.6**	**-0.89**
**RplU**	**PA4568**	**50S ribosomal protein L21**	**9**	**69.9**	**-0.89**
**ArgS**	**PA5051***	**arginyl-tRNA synthetase**	**1**	**29.6**	**-0.86**
**RpsJ**	**PA4264**	**30S ribosomal protein S10**	**9**	**57.3**	**-0.86**
**GroS**	**PA4386**	**co-chaperonin**	**7**	**60.8**	**-0.85**
MinE	PA3245	cell division topological specificity factor	3	32.1	-0.85
**RplC**	**PA4263**	**50S ribosomal protein L3**	**10**	**55.9**	**-0.81**
NqrF	PA2994	Na(+)-translocating NADH-quinone reductase subunit F	5	21.4	-0.74
PA4753	PA4753	hypothetical protein	3	27.9	-0.69
**ClpV1**	**PA0090**	**secretion protein**	**8**	**12.4**	**-0.52**
**HisE**	**PA5067**	**phosphoribosyl-ATP pyrophosphatase**	**2**	**18.9**	**1.03**
HslO	PA5193	heat shock protein 33	4	24.6	1.11
NqrA	PA2999	Na(+)-translocating NADH-quinone reductase subunit A	2	27.4	1.18
**TolQ**	**PA0969**	**translocation protein**	**8**	**32.0**	**1.19**
AhpF	PA0140	alkyl hydroperoxide reductase	3	8.6	1.20
PilG	PA0408	pilus biosynthesis/twitching motility protein	4	43.7	1.28
ThrB	PA5495	homoserine kinase	2	12.0	1.32
**HutU**	**PA5100**	**urocanate hydratase**	**16**	**42.0**	**1.38**

Supplementary Table 5. Inclusive list of metabolically relevant, *P. aeruginosa* proteins with significant increases (blue highlighted, ↑), significant decreases (red highlighted, ↓), and non-significant abundance changes (no highlights) during growth in CSP medium biofilm vs. planktonic cultures (see Supplementary Figure 2). Boldface proteins are common to both LCSP (Figure 5) and CSP (Supplementary Figure 2) media regardless of abundance. '*' indicates homologous protein KEGG ID.

Protein	KEGG ID	Description of Proteins in Supplementary Figure 2	Unique Peptides	Sequence Coverage %	$\log_2(x_{BF}/x_{Plank})$
BetB	PA5373	betaine aldehyde dehydrogenase	4	16.1	1.79 ↑
**CysD**	**PA4443**	**ATP sulfurylase small subunit**	**4**	**15.7**	**2.22** ↑
**HisE**	**PA5067**	**phosphoribosyl-ATP pyrophosphohydrolase**	**2**	**18.9**	**2.41** ↑
**PolA**	**PA5493**	**DNA polymerase I**	**5**	**11.3**	**2.69** ↑
CysA	PA0280	sulfate.thiosulfate ABC transporter ATP-binding protein	4	23.7	2.69 ↑
**OprD**	**PA0958**	**porin D**	**12**	**37.5**	**3.40** ↑
**FptA**	**PA4221**	**Fe(III)-pyochelin outer membrane receptor**	**40**	**70.4**	**7.35** ↑
**CcpA**	**PA4587**	**cytochrome C551 peroxidase**	**12**	**54.0**	**-8.42** ↓
HutH	PA5098	histidine ammonia-lyase	6	24.4	-5.24 ↓
RimO	PA0916	ribosomal protein S12 methylthiotransferase	3	15.7	-4.98 ↓
**NfuA**	**PA1847**	**Fe/S biogenesis protein**	**4**	**38.7**	**-4.60** ↓
LpdV	PA2250	branched-chain alpha-keto acid dehydrogenase complex dihydrolipoyl dehydrogenase	5	23.7	-4.23 ↓
NosZ	PA3392	nitrous-oxide reductase	5	12.9	-4.12 ↓
RplS	PA3742*	LSU ribosomal protein L19P	1	30.2	-4.00 ↓
**PA1673**	**PA1673**	**bacteriohemerythrin**	**5**	**45.1**	**-3.95** ↓
**TatA**	**PA5068**	**twin-arginine translocation protein**	**4**	**63.4**	**-3.80** ↓
**CoaD**	**PA0363**	**phosphopantetheine adenylyltransferase**	**3**	**23.9**	**-3.79** ↓
**OprI**	**PA2853**	**outer membrane lipoprotein**	**7**	**68.7**	**-3.72** ↓
**AcnA**	**PA1562**	**aconitate hydratase**	**30**	**51.2**	**-3.65** ↓
**RplL**	**PA4271**	**50S ribosomal protein L7/L12**	**9**	**86.1**	**-3.43** ↓
**SecY**	**PA4243**	**preprotein translocase subunit**	**4**	**10.4**	**-3.29** ↓
MurC	PA4411	UDP-N-acetylmuramate--L-alanine ligase	3	12.7	-3.08 ↓
ProC	PA0393	pyrroline-5-carboxylate reductase	3	16.1	-3.05 ↓
MtnD	PA1684	acireductone dioxygenase	2	23.2	-3.03 ↓
**PhhA**	**PA0872**	**phenylalanine 4-monooxygenase**	**4**	**17.9**	**-3.02** ↓
**OprF**	**PA1777**	**outer membrane porin F**	**14**	**40.0**	**-2.99** ↓
**SecG**	**PA4747**	**preprotein translocase subunit**	**2**	**37.2**	**-2.94** ↓
**Mqo1**	**PA3452**	**malate:quinone oxidoreductase**	**8**	**21.6**	**-2.91** ↓
**RpmD**	**PA4245**	**50S ribosomal protein L30**	**1**	**24.1**	**-2.86** ↓
**DnaK**	**PA4761***	**chaperone protein**	**4**	**19.9**	**-2.85** ↓
**NuoI**	**PA2644**	**NADH-quinone oxidoreductase subunit I**	**4**	**23.1**	**-2.77** ↓
PilB	PA4526	type 4 fimbrial biogenesis protein	3	10.6	-2.71 ↓
GcvH2	PA2446	glycine cleavage system protein H	2	15.7	-2.68 ↓
AcsA2	PA4733	acetyl-CoA synthetase	3	9.5	-2.58 ↓
**PA2953**	**PA2953**	**electron transfer flavoprotein-ubiquinone oxidoreductase**	**12**	**32.3**	**-2.58** ↓

Protein	KEGG ID	Description of Proteins in Supplementary Figure 2	Unique Peptides	Sequence Coverage %	$log_2(x_{BF}/x_{Plank})$
**RplT**	**PA2741**	**50S ribosomal protein L20**	**3**	**22.9**	**-2.52** ↓
PckA	PA5192	phosphoenolpyruvate carboxykinase	0	48.9	-2.51 ↓
**NirS**	**PA0519**	**nitrite reductase**	**13**	**33.1**	**-2.46** ↓
**KatA**	**PA4236**	**catalase**	**5**	**22.4**	**-2.46** ↓
HmgA	PA2009	homogentisate 1,2-dioxygenase	18	70.8	-2.43 ↓
GlmM	PA4749	phosphoglucosamine mutase	9	31.0	-2.38 ↓
Ung	PA0750	uracil-DNA glycosylase	6	39.4	-2.34 ↓
**HemN**	**PA1546**	**oxygen-independent coproporphyrinogen-III oxidase**	**10**	**34.3**	**-2.29** ↓
**ArcC**	**PA5173**	**carbamate kinase**	**15**	**50.6**	**-2.26** ↓
**NuoC**	**PA2639**	**NADH:-quinone oxidoreductase subunit C/D**	**18**	**39.5**	**-2.22** ↓
NadA	PA1004	quinolinate synthetase	2	13.9	-2.20 ↓
MurE	PA4417	UDP-N-acetylmuramoylalanyl-D-glutamate--2,6-diaminopimelate ligase	3	12.5	-2.19 ↓
**GlmU**	**PA5552**	**bifunctional glucosamine-1-phosphate acetyltransferase/N-acetylglucosamine-1-phosphate uridyltransferase**	**5**	**18.7**	**-2.19** ↓
**RplM**	**PA4433**	**50S ribosomal protein L13**	**6**	**54.9**	**-2.12** ↓
**CycH**	**PA1483**	**cytochrome c-type biogenesis protein**	**4**	**19.9**	**-2.12** ↓
**PagL**	**PA4661**	**lipid A 3-O-deacylase**	**3**	**22.0**	**-2.12** ↓
NusB	PA4052	transcription antitermination protein	3	39.6	-2.10 ↓
ArcB	PA5172	ornithine carbamoyltransferase	22	80.1	-2.06 ↓
ThrH	PA1757	phosphoserine phosphatase	3	26.3	-2.06 ↓
**RpmB**	**PA5316**	**50S ribosomal protein L28**	**5**	**52.6**	**-2.05** ↓
Fbp	PA5110	fructose-1,6-bisphosphatase	8	27.4	-2.05 ↓
**MtnB**	**PA1683**	**methylthioribulose-1-phosphate dehydratase**	**1**	**12.2**	**-2.05** ↓
**PA5475**	**PA5475**	**hypothetical protein**	**6**	**50.0**	**-2.03** ↓
DnaE	PA3640	DNA polymerase III subunit alpha	5	9.5	-2.03 ↓
ArcA	PA5171	arginine deiminase	29	72.0	-2.02 ↓
MucB	PA0764	sigma factor AlgU regulator	3	16.5	-2.01 ↓
**NuoG**	**PA2642**	**NADH-quinone oxidoreductase subunit G**	**24**	**40.8**	**-2.00** ↓
**DadA1**	**PA5304**	**D-amino acid dehydrogenase small subunit**	**3**	**8.8**	**-1.98** ↓
AspA	PA5429	aspartate ammonia-lyase	6	28.5	-1.97 ↓
SpeE1	PA1687	polyamine aminopropyltransferase	4	26.2	-1.94 ↓
**NuoE**	**PA2640**	**NADH-quinone oxidoreductase subunit E**	**5**	**44.6**	**-1.93** ↓
KdsD	PA4457	arabinose-5-phosphate isomerase	2	14.4	-1.91 ↓
SutA	PA5285*	conserved hypothetical protein	2	31.4	-1.88 ↓
AtoB	PA2001	acetyl-CoA acetyltransferase	12	50.6	-1.88 ↓
**Ssb**	**PA4232**	**single-stranded DNA-binding protein**	**5**	**37.0**	**-1.88** ↓
**IlvD**	**PA0353**	**dihydroxy-acid dehydratase**	**13**	**35.3**	**-1.88** ↓
**AcnB**	**PA1787**	**aconitate hydratase B**	**41**	**73.4**	**-1.87** ↓
GlyQ	PA0009	glycine--tRNA ligase subunit alpha	8	38.7	-1.86 ↓
**PurT**	**PA3751**	**phosphoribosylglycinamide formyltransferase**	**4**	**15.3**	**-1.81** ↓
**Frr**	**PA3653***	**ribosome recycling factor**	**1**	**10.3**	**-1.80** ↓

Protein	KEGG ID	Description of Proteins in Supplementary Figure 2	Unique Peptides	Sequence Coverage %	$\log_2(x_{BF}/x_{Plank})$
**RplB**	**PA4260**	**50S ribosomal protein L2**	**12**	**50.9**	**-1.80** ↓
**RpsS**	**PA4259**	**30S ribosomal protein S19**	**5**	**46.2**	**-1.79** ↓
**AccB**	**PA4847**	**acetyl-CoA carboxylase biotin carboxyl carrier protein subunit**	**4**	**53.2**	**-1.78** ↓
**AotP**	**PA0892**	**arginine/ornithine ABC transporter ATP-binding protein AotP**	**5**	**29.5**	**-1.75** ↓
PhhB	PA0871	pterin-4-alpha-carbinolamine dehydratase	7	78.8	-1.75 ↓
BauC	PA0130	3-oxopropanoate dehydrogenase	8	31.0	-1.74 ↓
UbiE	PA5063	ubiquinone/menaquinone biosynthesis methyltransferase	4	35.2	-1.72 ↓
Def	PA0019	peptide deformylase	3	21.4	-1.72 ↓
AguA	PA0292	agmatine deiminase	6	22.6	-1.71 ↓
MscL	PA4614	large-conductance mechanosensitive channel	7	51.1	-1.70 ↓
GlyA2	PA2444	serine hydroxymethyltransferase	4	20.3	-1.70 ↓
**NuoF**	**PA2641**	**NADH dehydrogenase I subunit F**	**11**	**40.4**	**-1.70** ↓
**RpmE**	**PA5049**	**50S ribosomal protein L31**	**3**	**71.8**	**-1.69** ↓
**SodB**	**PA4366**	**superoxide dismutase**	**6**	**58.0**	**-1.66** ↓
**NuoB**	**PA2638**	**NADH-quinone oxidoreductase subunit B**	**6**	**32.0**	**-1.66** ↓
**HemB**	**PA5243**	**delta-aminolevulinic acid dehydratase**	**6**	**37.1**	**-1.66** ↓
MurD	PA4414	UDP-N-acetylmuramoyl-L-alanyl-D-glutamate synthetase	5	13.6	-1.65 ↓
**FumC1**	**PA4470**	**fumarate hydratase**	**10**	**42.0**	**-1.65** ↓
AtpF	PA5558	ATP synthase subunit B	9	55.1	-1.63 ↓
GrpE	PA4762	heat shock protein	12	84.4	-1.62 ↓
**NagZ**	**PA3005**	**beta-hexosaminidase**	**2**	**11.7**	**-1.61** ↓
**RpsM**	**PA4241**	**30S ribosomal protein S13**	**9**	**70.3**	**-1.57** ↓
DavT	PA0266	5-aminovalerate aminotransferase	19	75.1	-1.54 ↓
AceF	PA5016	dihydrolipoamide acetyltransferase	22	60.0	-1.53 ↓
**RplR**	**PA4247**	**50S ribosomal protein L18**	**9**	**58.6**	**-1.52** ↓
RplX	PA4252	50S ribosomal protein L24	7	61.5	-1.51 ↓
SucB	PA1586	2-oxoglutarate dehydrogenase complex dihydrolipoyllysine-residue succinyltransferase	21	63.1	-1.49 ↓
**RpsL**	**PA4268**	**30S ribosomal protein S12**	**4**	**32.5**	**-1.48** ↓
DnaJ	PA4760	molecular chaperone	13	52.8	-1.48 ↓
**PmpR**	**PA0964**	**transcriptional regulator**	**3**	**25.0**	**-1.47** ↓
**PyrC**	**PA3527**	**dihydroorotase, homodimeric type**	**2**	**6.3**	**-3.09**
**Dxs**	**PA4044**	**1-deoxy-D-xylulose-5-phosphate synthase**	**2**	**8.1**	**-2.09**
GuaA	PA3769	GMP synthase	24	64.6	-1.44
AstB	PA0899	N-succinylarginine dihydrolase	11	39.7	-1.43
**DapD**	**PA3666***	**tetrahydrodipicolinate succinylase**	**1**	**39.2**	**-1.43**
PdxH	PA1049	pyridoxine/pyridoxamine 5'-phosphate oxidase	6	44.2	-1.43
NadE	PA4920	NAD synthetase	11	60.7	-1.42
MexA	PA0425	multidrug resistance protein	15	58.5	-1.42
MaiA	PA2007	maleylacetoacetate isomerase	5	39.2	-1.41

Protein	KEGG ID	Description of Proteins in Supplementary Figure 2	Unique Peptides	Sequence Coverage %	$\log_2(x_{BF}/x_{Plank})$
MurG	PA4412	undecaprenyldiphospho-muramoylpentapeptide beta-N-acetylglucosaminyltransferase	3	19.0	-1.40
EtfA	PA2951	electron transfer flavoprotein subunit alpha	13	80.3	-1.39
GatA	PA4483	Glu-tRNA(Gln) amidotransferase subunit A	18	55.6	-1.39
TyrS	PA4138	tyrosyl-tRNA synthetase	7	33.5	-1.39
PurM	PA0945	phosphoribosylformylglycinamidine cyclo-ligase	11	42.2	-1.38
FtsA	PA4408	cell division protein	14	47.0	-1.37
RplP	PA4256	50S ribosomal protein L16	7	48.2	-1.37
**PA2652**	**PA2652**	**methyl-accepting chemotaxis protein**	**7**	**20.1**	**-1.37**
**HutU**	**PA5100**	**urocanase**	**24**	**57.6**	**-1.37**
**RpsJ**	**PA4264**	**30S ribosomal protein S10**	**8**	**57.3**	**-1.36**
RplK	PA4274	50S ribosomal protein L11	7	51.7	-1.35
PanB2	PA4729	3-methyl-2-oxobutanoate hydroxymethyltransferase	7	44.0	-1.35
**GroS**	**PA4386**	**Hsp10 protein**	**2**	**60.8**	**-1.34**
**RplJ**	**PA4272**	**50S ribosomal protein L10**	**7**	**44.6**	**-1.34**
DavD	PA0265	glutarate-semialdehyde dehydrogenase	19	54.5	-1.33
FliC	n/a	A-type flagellin	18	59.9	-1.33
**ArgS**	**PA5051***	**arginyl-tRNA synthetase**	**1**	**23.7**	**-1.31**
**PheS**	**PA2740**	**phenylalanyl-tRNA synthetase, alpha-subunit**	**9**	**33.4**	**-1.31**
FtsZ	PA4407	cell division protein	13	40.1	-1.30
**RplV**	**PA4258**	**50S ribosomal protein L22**	**8**	**54.5**	**-1.30**
SerS	PA2612	serine--tRNA ligase	14	56.3	-1.29
CarB	PA4756	carbamoyl phosphate synthase large subunit	31	41.5	-1.27
**RplC**	**PA4263**	**50S ribosomal protein L3**	**8**	**41.2**	**-1.26**
GpsA	PA1614	glycerol-3-phosphate dehydrogenase, biosynthetic	5	19.1	-1.26
SthA	PA2991	soluble pyridine nucleotide transhydrogenase	13	45.7	-1.22
AccC	PA4848	acetyl-CoA carboxylase biotin carboxylase subunit	18	52.8	-1.21
RpsI	PA4432	30S ribosomal protein S9	5	36.9	-1.21
RpsQ	PA4254	30S ribosomal protein S17	6	60.2	-1.18
RibH	PA4053	6,7-dimethyl-8-ribityllumazine synthase	7	68.4	-1.17
PctB	PA4310	chemotactic transducer	5	41.3	-1.16
LeuS	PA3987	leucyl-tRNA synthetase	2	26.8	-1.16
ClpB	PA4542	chaperone protein	45	60.8	-1.16
AlaS	PA0903	alanine--tRNA ligase	14	31.7	-1.16
AckA	PA0836	acetate kinase	3	14.0	-1.15
GlnS	PA1794	glutaminyl-tRNA synthetase	20	45.0	-1.14
**RplU**	**PA4568**	**50S ribosomal protein L21**	**9**	**69.9**	**-1.14**
CarA	PA4758	carbamoyl phosphate synthase small subunit	6	30.7	-1.13
Adk	PA3686	adenylate kinase	13	78.1	-1.10
RplA	PA4273	50S ribosomal protein L1	13	57.6	-1.08
GltX	PA3134	glutamate--tRNA ligase	1	29.1	-1.08
ArgG	PA3525	argininosuccinate synthase	16	49.6	-1.07

Protein	KEGG ID	Description of Proteins in Supplementary Figure 2	Unique Peptides	Sequence Coverage %	$\log_2(x_{BF}/x_{Plank})$
**MutS**	**PA3620**	**DNA mismatch repair protein**	**6**	**9.8**	**-1.07**
HslV	PA5053	ATP-dependent protease peptidase subunit	3	19.2	-1.07
**Cc4**	**PA5490**	**cytochrome C4**	**4**	**33.8**	**-1.06**
Gap2	PA3001*	putative glyceraldehyde 3-phosphate dehydrogenas	19	51.8	-1.05
DapB	PA4759	dihydrodipicolinate reductase	6	36.9	-1.04
RibB	PA4054	3,4-dihydroxy-2-butanone-4-phosphate synthase	9	38.9	-1.04
AlgR	PA5261	alginate biosynthesis regulatory protein	8	53.6	-1.04
Pgi	PA4732	glucose-6-phosphate isomerase	8	21.1	-1.03
RpsG	PA4267	30S ribosomal protein S7	12	60.3	-1.02
HslU	PA5054	ATP-dependent protease ATP-binding subunit	7	40.3	-1.01
RecA	PA3617	RecA protein	14	59.0	-1.01
Hcp1	PA0085	protein secretion apparatus assembly protein	6	38.9	-0.99
PA0170	PA0170	hypothetical protein	2	28.6	-0.99
HemC	PA5260	porphobilinogen deaminase	6	28.8	-0.99
**ClpV1**	**PA0090**	**secretion protein**	**3**	**5.0**	**-0.98**
Eco	PA2755	ecotin precursor	6	46.2	-0.95
Psd	PA4957	phosphatidylserine decarboxylase	3	20.4	-0.94
GlyS	PA0008	glycyl-tRNA synthetase beta chain	16	38.2	-0.93
PurL	PA3763	phosphoribosylformylglycinamidine synthase	19	23.6	-0.91
TolR	PA0970	translocation protein	2	17.1	-0.91
PtsN	PA4464	nitrogen regulatory IIA protein	3	24.0	-0.91
Efp	PA2851	elongation factor P	7	37.8	-0.90
AruC	PA0895	acetylornithine aminotransferase	16	67.0	-0.89
PurH	PA4854	bifunctional phosphoribosylaminoimidazolecarboxamide formyltransferase/IMP cyclohydrolase	16	37.4	-0.89
LysS	PA3700	lysyl-tRNA synthetase	22	56.7	-0.87
IspG	PA3803	4-hydroxy-3-methylbut-2-en-1-yl diphosphate synthase (flavodoxin)	6	22.6	-0.87
ThrC	PA3735	threonine synthase	10	31.3	-0.86
**RpsK**	**PA4240**	**30S ribosomal protein S11**	**7**	**60.5**	**-0.85**
LtaE	PA5413	low specificity l-threonine aldolase	7	26.6	-0.85
PurD	PA4855	phosphoribosylamine--glycine ligase	8	27.3	-0.80
AtpC	PA5553	ATP synthase subunit epsilon	4	30.5	-0.80
GreA	PA4755	transcription elongation factor	9	67.1	-0.79
FabZ	PA3645	3-hydroxyacyl-[acyl-carrier-protein] dehydratase	4	43.8	-0.77
GalU	PA2023	UTP-glucose-1-phosphate uridylyltransferase	11	57.3	-0.76
InfB	PA4744	translation initiation factor IF-2	26	43.2	-0.76
PurC	PA1013	phosphoribosylaminoimidazole-succinocarboxamide synthase	6	51.7	-0.72
ThrS	PA2744	threonine--tRNA ligase	26	48.0	-0.71
CobO	PA1272	cob(I)yrinic acid a,c-diamide adenosyltransferase	2	9.9	-0.70
Dcd	PA3480	deoxycytidine triphosphate deaminase	3	18.6	-0.70
CoaX	PA4279	pantothenate kinase	2	11.3	-0.68
PA3286	PA3286	3-oxoacyl-ACP synthase	9	37.4	-0.66

Protein	KEGG ID	Description of Proteins in Supplementary Figure 2	Unique Peptides	Sequence Coverage %	$\log_2(x_{BF}/x_{Plank})$
**TolQ**	**PA0969**	**translocation protein**	**7**	**31.2**	**0.70**
Ggt	PA1338	gamma-glutamyltranspeptidase	11	33.6	0.90
RpmE2	PA3601	50S ribosomal protein L31 type B	1	25.3	1.21

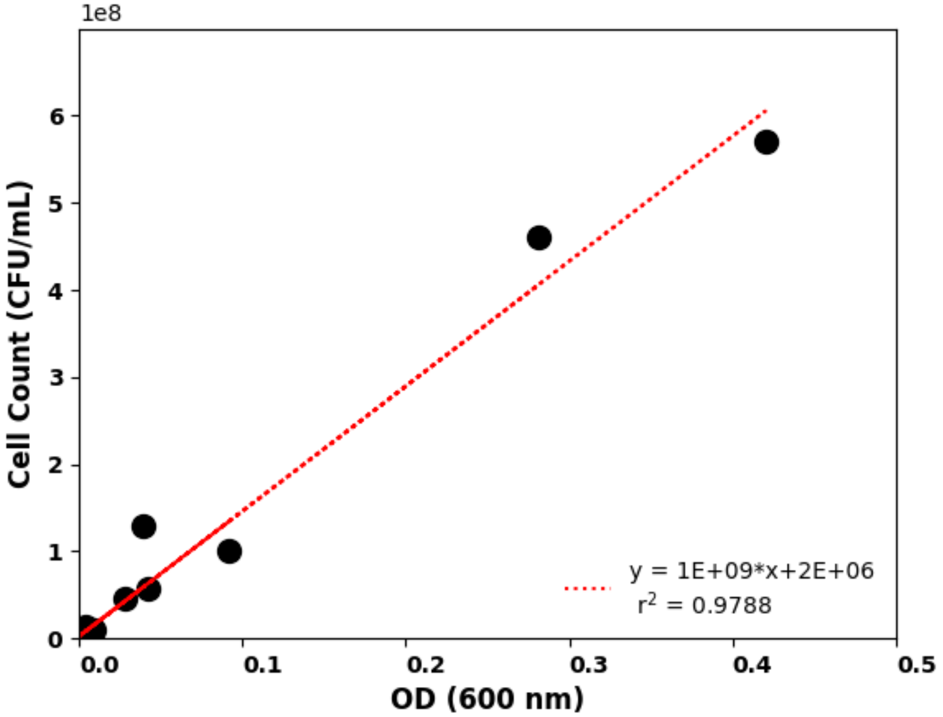

Supplementary Figure 1. Experimental correlation of *P. aeruginosa* isolate 215 optical density (OD 600 nm) and cell count (CFU/ml).

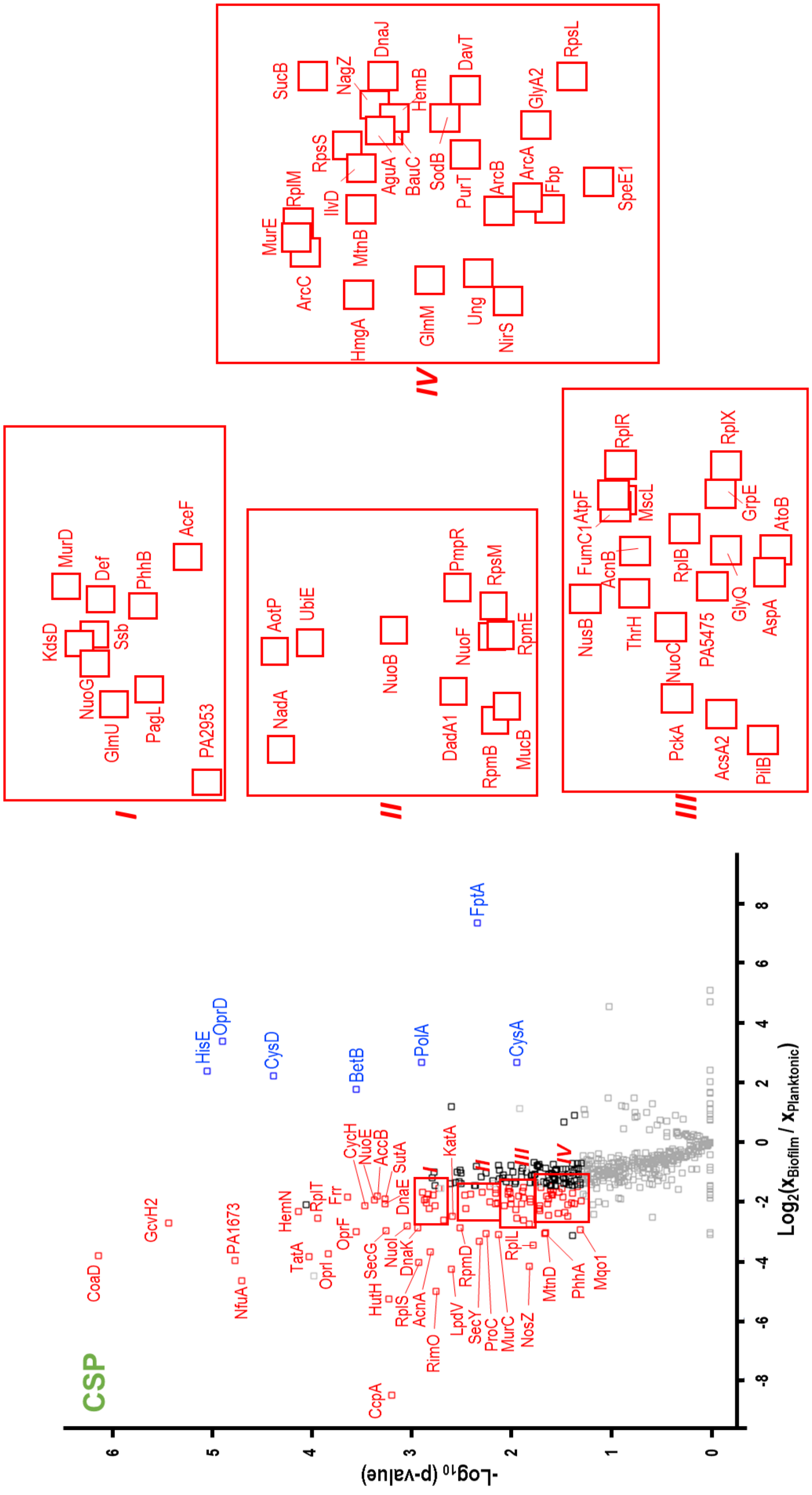

Supplementary Figure 2. *P. aeruginosa* protein abundance changes for biofilm vs. planktonic cultures grown on CSP medium for 11 h. Proteins with $|\log_2(x_{Biofilm}/x_{Planktonic})| > 1.5$ are deemed significantly lower or higher abundance and are annotated as red or blue points, respectively.

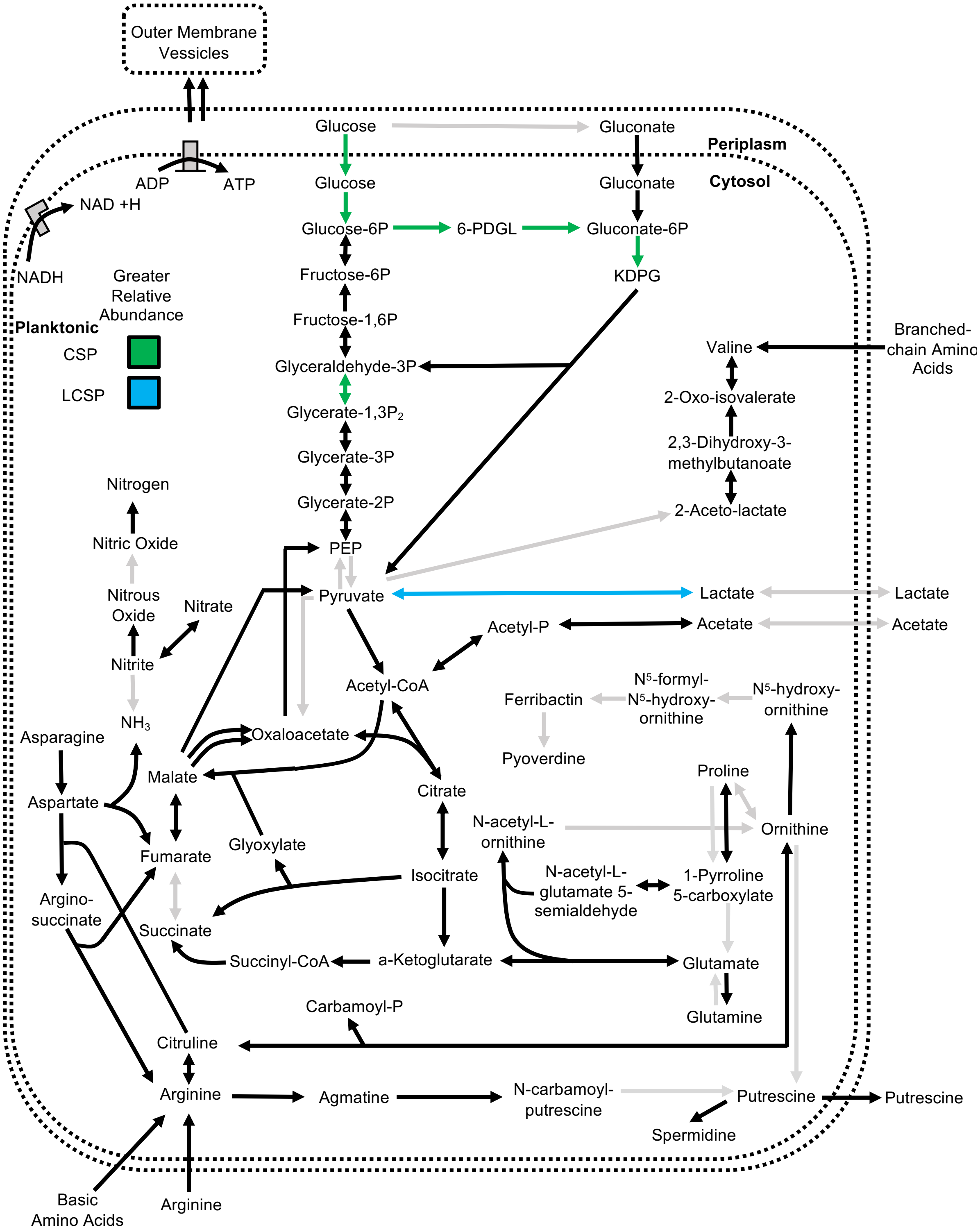

Supplementary Figure 3. Schematic of *P. aeruginosa* planktonic metabolism on CSP vs. LCSP. Dotted lines represent outer and inner cell membranes. Arrows represent proteins responsible for metabolic reactions. Black arrows represent proteins detected which do not have significant expression differentials. Grey arrows represent proteins which are known to exist in *P. aeruginosa* PAO1 but were not detected in this study. Green and blue arrows represent greater relative abundance of proteins from *P. aeruginosa* planktonic culture grown in CSP or LCSP, respectively.

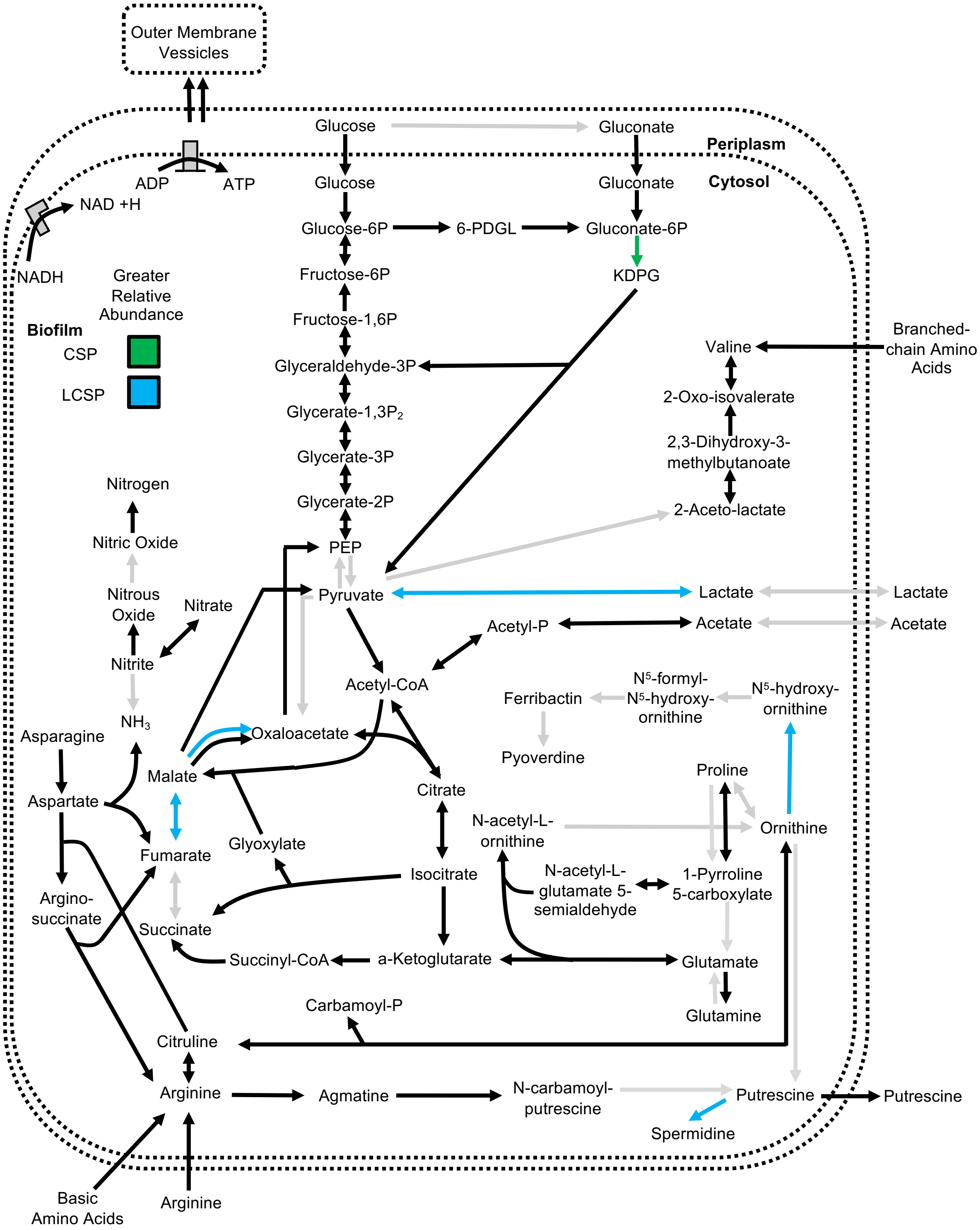

Supplementary Figure 4. Schematic of *P. aeruginosa* biofilm metabolism grown on CSP vs LCSP medium. Dotted lines represent outer and inner cell membranes. Arrows represent proteins responsible for metabolic reactions. Black arrows represent proteins detected which do not have significant expression differentials. Grey arrows represent proteins which are known to exist in *P. aeruginosa* PAO1 but were not detected in this study. Green and blue arrows represent greater relative abundance of proteins from *P. aeruginosa* cultures biofilm grown in CSP or LCSP, respectively.

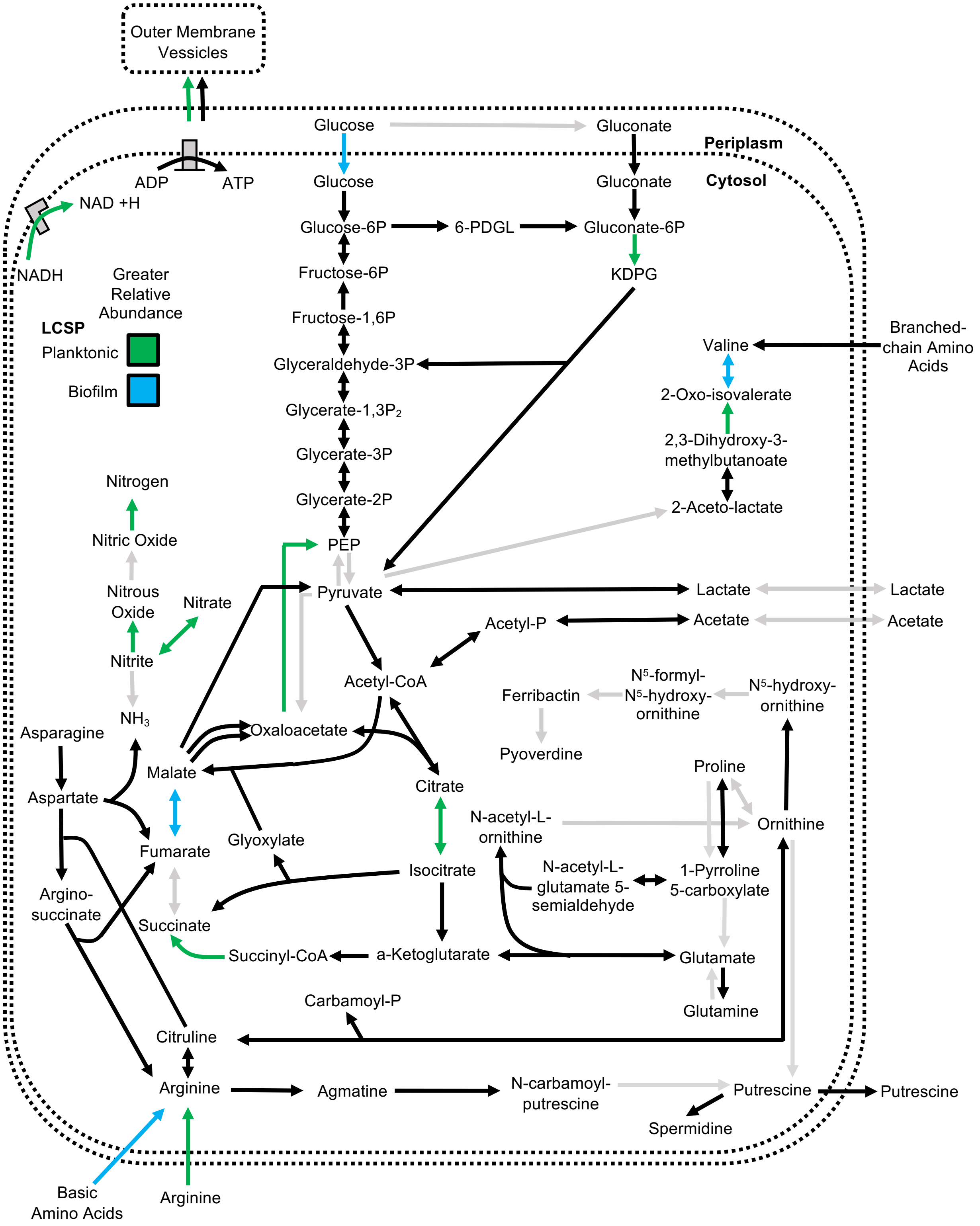

Supplementary Figure 5. Schematic of *P. aeruginosa* biofilm vs. planktonic metabolism in LCSP media. Dotted lines represent outer and inner cell membranes. Arrows represent proteins responsible for metabolic reactions. Black arrows represent proteins detected which do not have significant expression differentials. Grey arrows represent proteins which are known to exist in *P. aeruginosa* PAO1 but were not detected in this study. Green and blue arrows represent greater relative abundance of proteins from *P. aeruginosa* cultures grown in LCSP medium as planktonic or biofilm cultures, respectively.

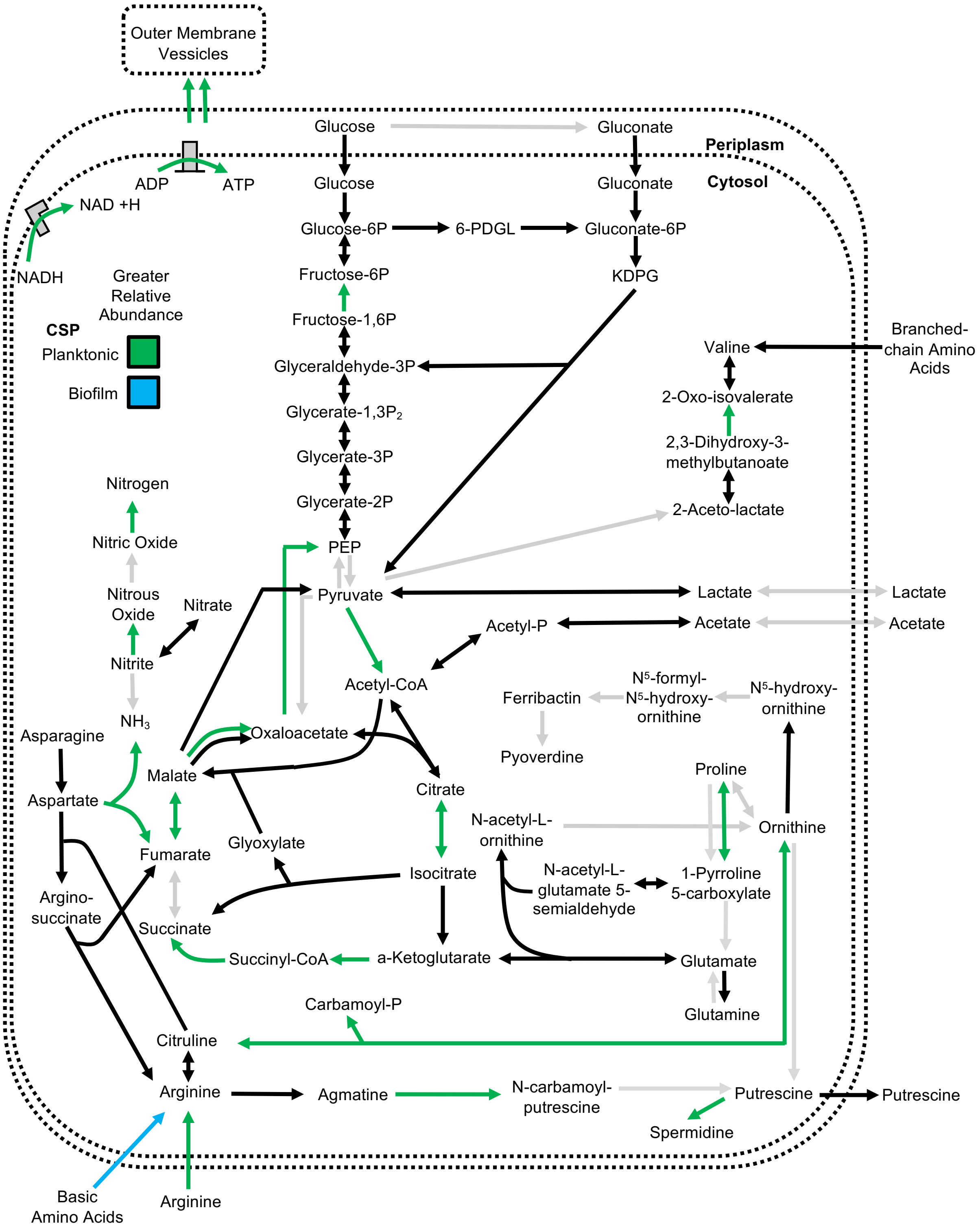

Supplementary Figure 6. Schematic of *P. aeruginosa* biofilm vs. planktonic metabolism in CSP media. Dotted lines represent outer and inner cell membranes. Arrows represent proteins responsible for metabolic reactions. Black arrows represent proteins detected which do not have significant expression differentials. Grey arrows represent proteins which are known to exist in *P. aeruginosa* PAO1 but were not detected in this study. Green and blue arrows represent greater relative abundance of proteins from *P. aeruginosa* cultures grown in CSP medium as planktonic or biofilm cultures, respectively.
